# Proteo-transcriptomics meta-analysis identifies SUMO2 as a promising target in glioblastoma multiforme therapeutics

**DOI:** 10.1186/s12935-021-02279-y

**Published:** 2021-10-29

**Authors:** Aswani P. Krishna, Sebastian John, Puja Laxmanrao Shinde, Rashmi Mishra

**Affiliations:** 1 Brain and Cerebro-Vascular Mechanobiology Research, Laboratory of Translational Mechanobiology, Department of Neurobiology, Rajiv Gandhi Centre for Biotechnology, Thiruvananthapuram, 695014 Kerala India; 2 Manipal Academy of Higher Education, Manipal, Karnataka India

**Keywords:** Glioblastoma multiforme, Cancer drug target discovery, SUMO2, Kinases and cancer, TCGA

## Abstract

**Background:**

Glioblastoma multiforme (GBM) is a deadly brain tumour with minimal survival rates due to the ever-expanding heterogeneity, chemo and radioresistance. Kinases are known to crucially drive GBM pathology; however, a rationale therapeutic combination that can simultaneously inhibit multiple kinases has not yet emerged successfully.

**Results:**

Here, we analyzed the GBM patient data from several publicly available repositories and deduced hub GBM kinases, most of which were identified to be SUMOylated by SUMO2/3 isoforms. Not only the hub kinases but a significant proportion of GBM upregulated genes involved in proliferation, metastasis, invasion, epithelial-mesenchymal transition, stemness, DNA repair, stromal and macrophages maintenance were also identified to be the targets of SUMO2 isoform. Correlatively, high expression of SUMO2 isoform was found to be significantly associated with poor patient survival.

**Conclusions:**

Although many natural products and drugs are evidenced to target general SUMOylation, however, our meta-analysis strongly calls for the need to design SUMO2/3 or even better SUMO2 specific inhibitors and also explore the SUMO2 transcription inhibitors for universally potential, physiologically non-toxic anti-GBM drug therapy.

**Graphical Abstract:**

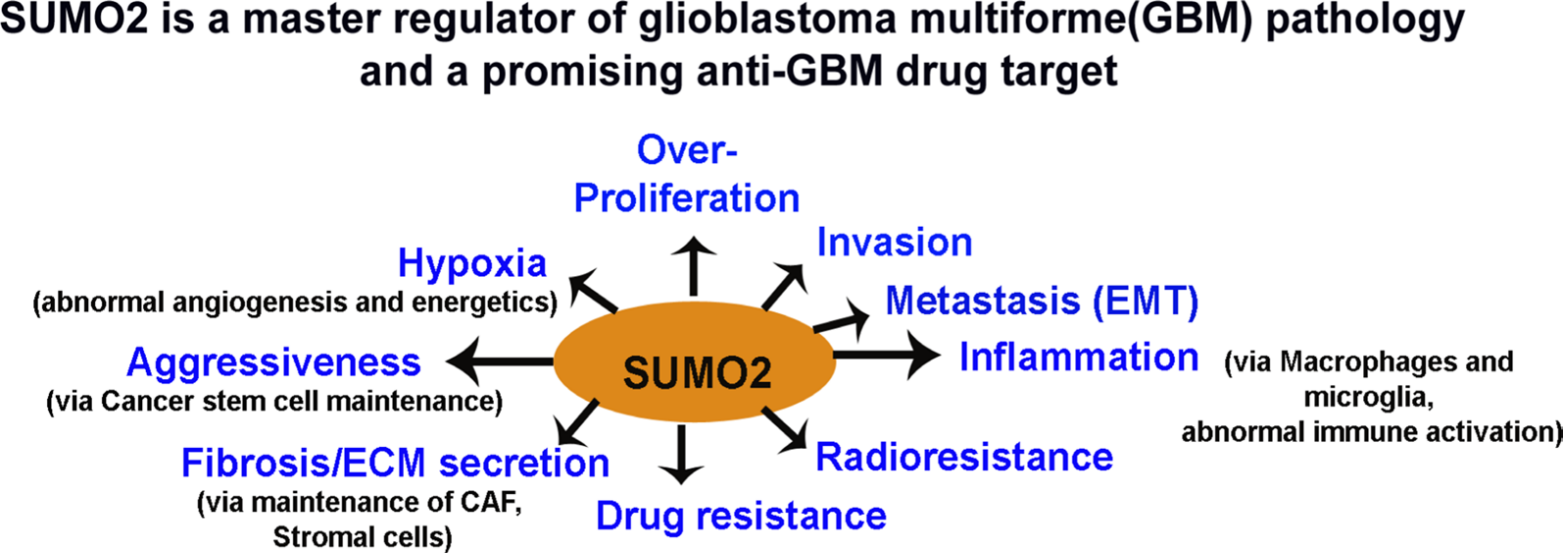

**Supplementary Information:**

The online version contains supplementary material available at 10.1186/s12935-021-02279-y.

## Highlights

The major highlights of this study are as follows:

- Key upregulated hub kinases and coding genes in GBM are found to be targets of SUMO2 conjugation.
- SUMO2 is significantly expressed in adult primary and recurrent GBMs as well as in pediatric GBM tumours.
- Orthotropic xenografts from adult and pediatric GBMs confirm high expression of SUMO2 in GBM tumour samples.
- SUMO2 is significantly associated with patient survival plot and pan-cancer cell fitness.
- Rationale design of SUMO2 inhibitors or search for its transcriptional inhibitors is urgently required through industry-academia collaboration for an anti-GBM and potentially pan-cancer therapeutics.

**Supplementary Information:**

The online version contains supplementary material available at 10.1186/s12935-021-02279-y.

## Background

Glioblastoma multiforme (GBM) is an astrocyte-derived brain tumour with minimal survival rates. It is the deadliest of all cancers thwarted with dismal hopes of any rationale therapeutics due to the scale and complexity of pathology across ages, genders, ethnicities and intracellular plasticity [1]. While basic laboratory research through the “in vitro*”* cell culture studies and “in vivo*”* animal model screening of candidate drugs can lead to anti-GBM drug target discovery, such endeavours are most often not translatable in the clinical settings. The excavation of cancer cell-specific vulnerabilities and their progression into clinical utility is thus essentially required for GBM management. In this direction, kinases are identified as central players in the formation, maintenance and recurrence of aggressive GBM tumours [1]. Both receptor kinases and non-receptor kinases are expressed in GBMs such as EGFR/mutant EGFRvIII, IGFR, PDGFR, VEGFR, FGFR, TGFβR, mutant PTEN, PI3K, AKT, MAPK, mTOR, Fyn, Src kinases (c-Src, Yes, Lyn and Lck), GSK3, Serum glucocorticoid kinases (SGK), ATM, casein kinase 2 (CK2), focal adhesion kinases (FAKs), sphingosine kinases (SPHK), Ephrins, polo-like kinases (PLK1), Aurora kinases (AURKA). Cyclin-dependent kinases (CDKs), LIMK1/2, ROCK1/2, PKD1/2, PAKs, to name a few [1–3].

Post-translational modifications in kinases crucially regulate their activity. Since several kinases are activated by phosphorylation, the attempts are on to target phosphorylation sites. However, multiple phosphorylation sites (identified via phosphoproteomics) and de novo mutations in phosphorylation sites are posing enormous difficulties in identifying druggable site-specific inhibitors [4]. Besides, the rapid emergence of various somatic variants of these kinases brings forth another level of complexity in drug target site identification [5].

Moreover, multiple kinases need to be targeted simultaneously because there is a redundancy in their functions. For example, the PI3K/AKT/mTOR (PAM) pathway contributes significantly to oncogenesis and tumour progression. Multiple receptor tyrosine kinases such as EGFR/ EGFRvIII/IGFR/PDGFR generate redundant activation of phosphoinositide-3′-kinase (PI3K) signalling [6]. Similarly, inhibition of mTOR signaling transactivates MAPK, which is linked to PI3K activation and can produce redundant functions [7, 8].

Other than multisite-phosphorylations, SUMOylation of kinases is crucially observed in various pathologies, especially in cancers. SUMO-conjugation of proteins has been evidenced to help cancer cells cope with microenvironment and metabolic stresses, thereby enabling survival [9, 10]. GBMs are evidenced to have prominent SUMOylation of both receptor and non-receptor kinases and other protumourigenic coding genes [11–13]. SUMOylation of CDK6, various cyclins, PI3K, AKT, ERK5 drive cell cycle progression and proliferation in GBMs [13]. SUMOylation of several cytoskeletal proteins such as vimentin and collapsin response mediator protein 2 (CRMP2) are also reported to promote over-proliferation [14, 15]. SUMOylation of ERK5, IGFR and EGFR allows their trafficking to the nucleus, where they upregulate pro-proliferative cyclin D1 and beta-catenin transcription factors [16–18]. Many G2/M checkpoint kinases such as BUB1B/BubR1, MPS1/TTK, Aurora-B, polo-like kinase 1 (PLK) are modified by SUMOylation, only upon which they serve to promote mitotic progression and cell division [10]. SUMOylation of Lats1 and AMPK antagonizes their tumour-suppressor functions [19, 20], whereas SUMO modification of NPM-ALK and Erythropoietin-producing hepatocellular carcinoma (Eph) family of kinases promote anchorage-independent growth [10].

However, since SUMOylation is also essential for maintaining cell homeostasis, a general inhibition of SUMOylation is not desirable. We, therefore, wanted to explore, in a clinical setting, the relevance of targeting a single SUMO isoform in suppressing multiple protumourigenic kinases and other genes vs global SUMOylation. This is required because general SUMOylation inhibitors like ginkgolic and anacardic acid fail to decrease global SUMOylation in GBM cell lines [10].

Meta-analysis and research weaving of clinical cancer data available in different public repositories and publications enables connecting the dots and bringing together an innovative description of cancer cell druggable vulnerabilities than is usually possible in a single study [21]. Hence, these computational approaches can allow unique opportunities to prioritise “go versus no-go” decisions in developing bench-to-bedside approaches to conquer cancer cells. Therefore, we were motivated to utilize these bioinformatics approaches to explore if any SUMO isoform post-translation modifications can be targeted, in place of phosphorylation, to suppress the pro-tumorigenic functions of multiple GBM kinases.

## Materials and methods

The Additional Tables/Additional files section provides detailed information on additional methods, individual data points, analyzed datasets, and website links associated with bioinformatics data analysis.

### Data preparation

In this study, a non-redundant list of 723 kinases was created from various literature sources [22, 23]. The level 3 gene transcriptome profile data (RNAseq-HTSeq-Count) of 173 clinical samples of GDC TCGA Glioblastoma and associated metadata files were downloaded from the UCSC XENA browser (https://xenabrowser.net/datapages/?dataset=TCGA-GBM.htseq_counts.tsv&host=https%3A%2F%2Fgdc.xenahubs.net&removeHub=https%3A%2F%2Fxena.treehouse.gi.ucsc.edu%3A443) 07-19-2019 version [24]. UCSC XENA has mRNA HTSeq counts normalized across the samples and is log2(Count + 1) transformed. Samples of solid normal tissue, primary tumour, and recurrent tumour were chosen for analysis (https://gdc-hub.s3.us-east-1.amazonaws.com/download/TCGA-GBM.GDC_phenotype.tsv.gz; https://docs.gdc.cancer.gov/Data_Dictionary/viewer/#?view=table-entity-listanchor=clinical). Please see Additional file 1: Table S1 for clinical data, and Additional file 2: Table S1 for mRNA HTSeq counts data of patient samples. Raw count matrix was obtained by back transforming the log2(Count + 1) data using R code for differential expression analysis (Additional file 3: Table S1). R code for back conversion is provided in the additional methods.

### Identifying differentially expressed genes (DEGs)

Differential expression analysis was carried out with the DESeq2 package (version 1.24.0) in R Studio. Due to the high sensitivity and precision offered by the DESeq2 method, it is used for comparative analysis of transcriptomics data between test and control samples to obtain differentially expressed genes [25]. Rows with low count genes (counts < 10) were pre-filtered to reduce the memory size and to increase the speed of transformation and testing functions within DESeq2. Genes with *P*adj < 0.05 and FC >  = 2 were considered to be statistically significant DEGs. Differential expression analysis was performed for 3 sample types and three conditions, including primary tumour vs solid tissue normal, recurrent tumour vs solid tissue normal, and primary tumour vs recurrent tumour. From the kinases genes list, differentially expressed genes in all three conditions were manually curated.

### Visualizing DEG’s with Heatmap and Volcano plot

Significantly expressed kinases in the three conditions (primary GBM vs adjacent normal samples, recurrent GBM vs adjacent normal samples and primary GBM vs recurrent GBM samples) were categorized into up-regulated (log2FC >  = 1) and down-regulated (log2FC <  = − 1) genes. Heatmap visualization of the high and low expressed kinases in all three conditions and the volcano plots were created using the Morpheus tool from Broad Institute (https://software.broadinstitute.org/morpheus/) and Graphpad Prism (version6.01), respectively.

### Protein–protein interaction (PPI) network construction and hub genes selection

The Search Tool for the Retrieval of Interacting Genes (STRING version 11.0, https://string-db.org/) database is used to construct a PPI network with the predicted association for a group of genes [26]. String predicts protein–protein association, which includes both physical and functional interactions. It provides the protein functional association ranked by a confidence score. Cytoscape software (version3.7.1) was used for PPI graphical network visualization. In the network, each node represents a gene/protein and edges represent the connection between them. Cytoscape is a freely available software package, majorly used for visualizing and analysing molecular & genetic interaction networks [27].Cytoscape support many algorithms for the network layout representation and many plugins for further network analysis. We used an Edge-weighted Spring Embedded layout for our network and Network Analyzer to efficiently compute the topological network parameters like degree and betweenness centrality [28].

### Identifying hub genes from the PPI network

Cytohubba (version 0.1) plugin was used to get the top 30 hubs genes and sub-networks from the PPI network [29]. Cytohubba uses different algorithms to identify the sub-network of the hub genes from a more extensive network, which includes Density of Maximum Neighbourhood Component (DMNC), Maximum Neighbourhood Component (MNC), Maximal Clique Centrality (MCC) and degree. These methods are local based methods that consider the neighborhood of a node. The result from DMNC methods, however, did not show consistency when compared to the other three methods. So the top 30 genes obtained from three methods MNC, MCC and degree, were overlapped, and the overlapping set of genes were considered as major hubs.

### Gene set enrichment analysis

Gene set enrichment analysis was performed using GSEA software from Broad institute (version 4.0.3) [https://www.gsea-msigdb.org/gsea/index.jsp] [30]. GSEA Pre ranked method was used to understand the enriched pathways and functional annotation of ranked differentially expressed genes. Upregulated and downregulated pre-ranked kinase genes from DESeq2 analysis was used for GSEA analysis. Curated geneset databases “c2.all.v7.1.symbols.gmt”and “Human_Symbol_with_Remapping_MSigDB.v7.1.‌chip” were set as chip platform for pathway enrichment analysis, also “c8.all.v7.2.symbols” [Cell type signature gene sets] was used to retrieve curated cluster markers for cell types identified in single-cell sequencing studies of human tissues using GSEA software (version 4.0.3). After 1000 permutations, Normalized enrichment score (NES) was calculated and gene set with *p*-value < 0.05, FDR < 0.25 were considered significant. Functional enrichment analysis was performed using DAVID [version 6.8, https://david.ncifcrf.gov/] [31]], and gene ontology over-representation analysis was performed with clusterProfiler [version 3.18] [32].

### Survival analysis

Survival analysis was performed using the PrognoScan database [http://www.prognoscan.org/; http://dna00.bio.kyutech.ac.jp/PrognoScan/] [33]. The correlation between SUMO isoforms expression and survival in brain cancer patients were analysed, using PrognoScan and the Kaplan–Meier plot. The survival curves were plotted for high expression (red) and low expression (blue) groups dichotomized at the optimal cut-point. The significant corrected *p*-value threshold was adjusted at < 0.05.

### Cell fitness analysis

Pan-cancer SUMO isoforms associated with cell fitness data were collected from the Cancer Dependency Map Dataset [https://depmap.org/portal/depmap/] [34]. It comprises data of genome-wide CRISPR and shRNA screens to identify essential genes across hundreds of cancer cell lines.

### Protein expression analysis from the human protein atlas (HPA)

The human protein atlas (https://www.proteinatlas.org/) provides a complete resource of proteomics and transcriptomics data generated from antibody-based microarray profiling and RNA sequencing. It can be majorly used to study protein co-localization and expression in human tissues and cells. HPAnalyze version 3.12, a freely available R package, was used to retrieve and visualize the data from the human protein atlas. hpaVisPatho() function from HPAnalyze was used to visualize the expression of the protein of interest in each cancer [35].

### Other data download web links and data reference IDs used in the study

(i) UCSC Xena browser to compare TCGA tumour samples to GTEx normal samples to see if our gene or transcript is up- or down-regulated in one or more cancer types [https://xena.ucsc.edu/compare-tissue/]; (ii) Cancer Cell Line Encyclopedia (CCLE) [https://portals.broadinstitute.org/ccle]; (iii) RNA Seq data on subtype-specific differences in molecular and cellular composition at the margins of glioblastoma [Ref id: GEO-GSE59612, https://www.ncbi.nlm.nih.gov/geo/query/acc.cgi?acc=GSE59612, PMID-25114226]; (iv) microarray data from GBM samples of patients showing radioresistance and chemoresistance [Ref id: GEO-GSE7696, https://www.ncbi.nlm.nih.gov/geo/query/acc.cgi?acc=GSE7696, PMID-18565887, 21642372]; (iv) microarray data from the human glioblastoma cell culture resource (HGCC) [Ref id: GEO-GSE72217, https://www.hgcc.se/,PMID-26629530]; (vi) microarray data from patient-derived cell line and xenograft models of proneural, classical and mesenchymal glioblastoma [Ref id: GEO-GSE118793, https://www.ncbi.nlm.nih.gov/geo/query/acc.cgi?acc=GSE118793; Ref id:, SRA-PRJNA508446, https://www.ncbi.nlm.nih.gov/bioproject/PRJNA508446/]; (vii) pediatric high and low grade glioma; [CBTTC,https://xenabrowser.net/datapages/?cohort=Pediatric%20Brain%20Tumour%20Atlas%3A%20CBTTC&removeHub=https%3A%2F% 2Fxena.treehouse.gi.ucsc.edu%3A443]; (viii) adult high and low grade glioma [UCSC Xena, High grade glioma:https://gdc-hub.s3.us-east-1.amazonaws.com/download/TCGA-GBM.htseq_counts.tsv.gz; Low grade glioma:https://gdc-hub.s3.us-east-1.amazonaws.com/download/TCGA-LGG.htseq_counts.tsv.gz]; (ix) microarray data for gene expression in immune cells [https://joycelab.shinyapps.io/braintime/]; (x) GBM RNA Seq data on cancer stem cells from IVYGAP [https://glioblastoma.alleninstitute.org/, https://glioblastoma.alleninstitute.org/api/v2/well_known_file_download/305873915]; (xi) protein mass spectrometry data from PDC commons [https://proteomic.datacommons.cancer.gov/pdc/https://proteomic.datacommons.cancer.gov/pdc/study/PDC000204]; (xii) RNA Seq data of developing/prenatal human brain [https://portal.brain-map.org/http://www.brainspan.org/static/download.html]; (xiii) microarray data from patient-derived pediatric brain tumour cell lines and tumour animal models [Ref id: GEO-GSE99961, https://www.ncbi.nlm.nih.gov/geo/query/acc.cgi?acc=GSE99961].

## Results

### SUMO2 putatively modify upregulated kinases and coding genes to enable glioblastoma multiforme (GBM) pathology

Towards the inception of the multi-kinase targeting strategy for GBM, we first compiled a non-redundant list of 723 human kinases from various literature sources (Additional file 4: Table S1).

Next, to identify the expression of these kinases in GBM patients' tissue, the RNA-Seq samples from 168 GBM tumours (primary GBM n = 155, recurrent GBM n = 13) and 5 normal brain samples were downloaded from the UCSC XENA browser. The clinical data demonstrating the heterogeneity of the GBM samples is provided in the Additional file 1: Table S1.

Samples of solid normal tissues (non tumour adjacent reference sample), primary GBM tumours, and recurrent GBM tumours were chosen for differential transcriptomics analysis of GBM (Additional file 4: Table S2). Fold change FC ≥ 2, i.e., up-regulated (log2FC >  = 1) and down-regulated (log2FC <  = − 1) genes were considered to be statistically significant to the reference samples for all genes and kinases expression analysis (Additional file 4: Table S2-S4, Additional file 5: Figures S1, S2).

**Fig. 1:**
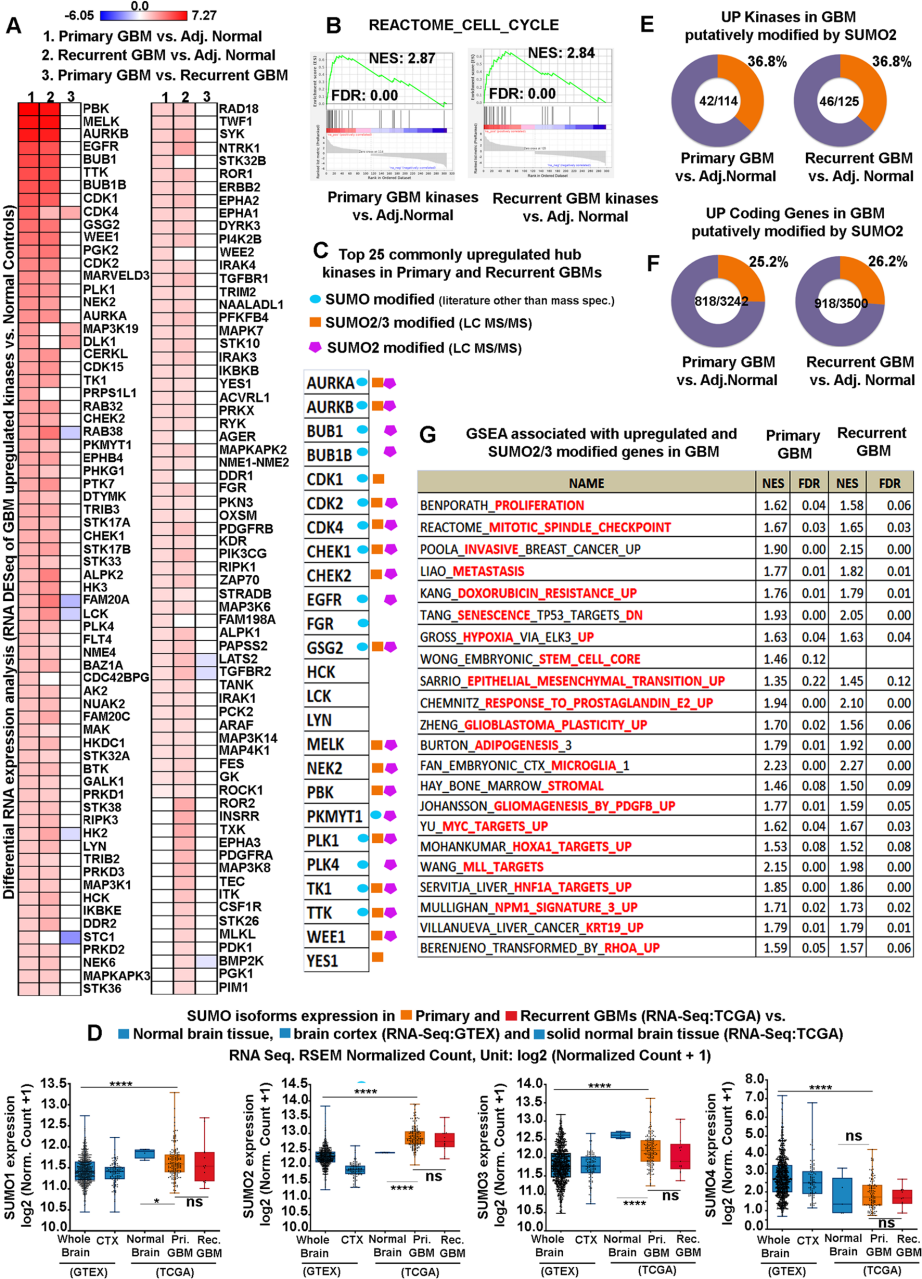
Glioblastoma upregulated kinases and coding genes are targets of SUMO2/3 isoforms.** A** Heatmap of RNA-Seq transcriptome analysis shows comparable upregulation of kinases in primary and recurrent glioblastoma tissue from 168 patients vs normal adjacent control tissues. The colour bar corresponds to per-gene log2 fold change values in compared groups. **B** Enrichment score plot/Gene set enrichment analysis (GSEA) identifies that upregulated kinases are predominantly involved in promoting the cell cycle. FDR = False Discovery Rate; FDR *q* value ≤ 0.25, NES = Normalized Enrichment score. Gene sets are ranked according to their normalized enrichment score (NES). NOM *p* value ≤ 0.05 was considered to be significant in sorting enriched terms. **C** CytoHubba network analysis of upregulated kinases in primary and recurrent GBMs unveils 25 common hub kinases amongst the top 30 hubs identified in individual networks. Mass spectrometry data from Hendriks et al. (2017 and 2018) identified 19 and 16 hub kinases as targets of SUMO2/3 (marked as an orange square) and SUMO2 (marked as a magenta polygon), respectively. Combined literature search (blue oval) and mass spectrometry data on SUMO2/3 targets amongst the top 25 common hub kinases in primary and recurrent GBMs identified 22 kinases to be targets of SUMO2/3. **D** A comparison of SUMO isoforms expression in normal whole brain and cerebral cortex alone [RNA Seq dataset from normal tissue (GTEx) gene expression dataset] with primary and recurrent GBM (RNA Seq dataset from TCGA) unveils significant expression of SUMO 1,2 and 3 isoforms. All datasets are reported as mean ± SD. *p < 0.05, **p < 0.01, ***p < 0.001 and ****p < 0.0001; Mean is derived from a statistically significant number of samples, and Student t-test function was used to drive significance. **E**, **F** Analysis of Hendriks et al., 2017 mass spectrometry-based proteomics data revealed that a substantial percentage of GBM upregulated kinases and coding genes are targets of SUMO2 isoform (represented as an orange colour fraction in the pie chart). **G** GSEA analysis of upregulated genes in primary and recurrent GBMs that are identified as targets of SUMO2/3 (via Hendriks LC–MS/MS data) showed enrichment in all hallmarks of cancers-proliferation, metastasis, invasion, EMT, hypoxia, drug resistance, pro-tumourigenic immune cell activation etc. For in-depth details on individual data points and dataset sample size in each graph, please refer to corresponding Additional tables mentioned in the main manuscript text

A total of 114 kinases were identified to be upregulated in primary tumours and 125 in recurrent tumours in comparison to the adjacent normal tissues (Fig. 1A and Additional file 4: Table S5). A comparison of upregulated kinases in primary GBMs and recurrent GBMs showed 102 commonly upregulated kinases (Fig. 1A, Additional file 4: Table S5). Functional gene set enrichment analysis (GSEA) of the upregulated kinases in primary and recurrent GBM samples were intriguingly associated with cell cycle and mitosis promoting processes (Fig. 1B and Additional file 4: Table S6). The top 30 differentially upregulated hub kinases were then screened in primary and recurrent samples (vs normal adjacent samples) by combining the three local‑based methods (MNC, MCC and degree) in the Cytoscape plugin cytoHubba (Additional file 4: Table S7). From the results obtained, 25 hub upregulated kinases were common in primary and recurrent GBM samples amongst the top 30 hubs obtained by individual methods (Fig. 1C and Additional file 4: Table S7).

RNA-Seq analysis showed high SUMO1, 2 and 3 isoforms in both primary and recurrent GBM tissue samples compared to unmatched normal brain control samples and tumour adjacent normal samples (Fig. 1D, Additional file 4: Table S8). We found that 88 percent [22 out of 25] of common hub kinases between primary and recurrent GBMs bear a capacity to be SUMOylated predominantly by SUMO2/3 isoforms (combined inferences from literature survey and mass-spectrometry studies) [Fig. 1C and Additional file 4: Table S9–11] [36].

Mass spectrometry data extraction confirmed 76% (19 out of 25) kinases as the targets of SUMO2 isoforms [Fig. 1C, Additional file 4: Table S12] [9]. Not only hubs but overall, approximately more than 30% percent of upregulated kinases in primary and recurrent GBM samples showed potential to be modified by SUMO2/3 via mass-spectrometry based analysis (Additional file 5: Figure S3A and Additional file 4: Table S13).

Not just kinases, approximately 19% of all upregulated coding genes were identified to bear a potential to be modified by SUMO2/3 isoforms (Additional file 4: Table S14 and Additional file 5: Figure S3B). SUMO2 directed mass spectrometry data showed 36% of all upregulated kinases and 25% of all upregulated coding genes to be the targets of SUMO2 conjugation (Fig. 1E and F, Additional file 4: Table S15 and S16).

The GSEA analysis of all upregulated GBM (primary and recurrent) genes putatively modified by SUMO2/3, showed enrichment for major pro-tumourigenic processes such as invasion, metastasis, proliferation, stemness, EMT, drug resistance, hypoxia, plasticity, downregulation of senescence, adipogenesis, upregulation of protumourigenic genes associated with HOX, Myc, MLL, HNFIA, NPM1, KRT19 and RhoA and enrichment of inflammatory stromal and microglial cells (Fig. 1G and Additional file 4: Table S17).

Besides, supplementary platforms for identification of gene enrichment processes such as DAVID GO Clusters and Over-representation tests showed an increase in DNA repair, protumourigenic sonic hedgehog, src kinase, Hox9, cyclophilin, oxidative stress response and folic acid metabolism pathways (Additional file 4: Table S18, 19, Additional file 5: Figures S4 and S5). Hence, broadly putative SUMO2/3 modification of kinases and other upregulated genes could be strongly associated with primary and recurrent GBM maintenance and progression.

Indeed, even the proteomics data showed high upregulation of SUMO2 and SUMO2/3 as analyzed through Protein Data Commons and Human protein Atlas respectively (Additional file 4: Tables S20, 21, Additional file 5: Figure S6).

### SUMO2 is significantly expressed in heterogenous cell types of GBM and is associated with poor prognosis

**Fig. 2:**
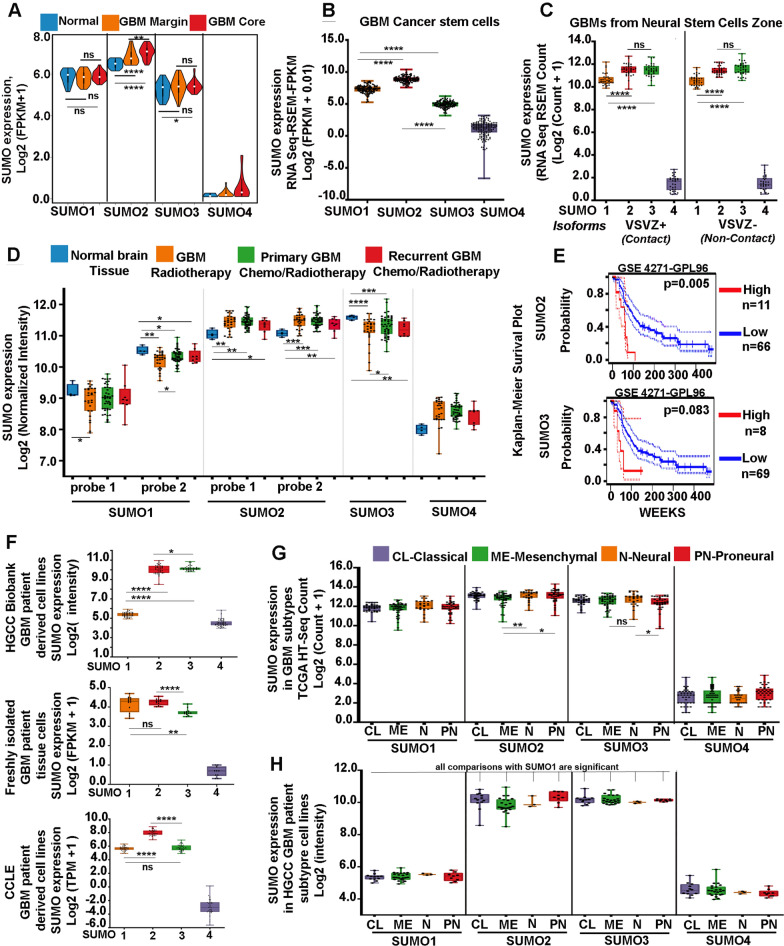
SUMO2 isoform is highly expressed across glioblastoma heterogeneity: **A** RNA Seq data (GEO-GSE59612) analysis from glioblastoma patients' tumour samples revealed that the whole glioblastoma tumour, that is, both core and margin (including residual tumour cells left behind after surgical resection) expresses significant levels of SUMO2 isoforms vs normal controls and other SUMO isoforms. **B** Glioblastoma cancer stem cells (CSCs) RNA Seq data analysis from the Allen Brain Atlas repository (IVY-GAP) reveals that CSCs are significantly enriched in SUMO2 isoforms vs other isoforms. **C** RNA Seq data analysis of human glioblastomas that were in contact with neural stem cell zones of the ventricular sub-ventricular regions (VSVZ +) in the brain as well as glioblastomas that were not in VSVZ contact (VSVZ-, non-contact) showed high SUMO2/3 expression vs other isoforms. VSVZ refers to the Ventricular Subventricular zone neural stem cell niche. Plus sign refers to glioblastomas in contact with ventricular-subventricular neural stem cell regions. VSVZ contact by GBMs has been noted for negatively impacting patient survival. Minus sign refers to glioblastoma bulk populations that did not contact ventricular neural stem cell lining, hence are in a different microenvironment. **D** Microarray data (GEO-GSE7696) analysis from human primary and recurrent glioblastoma tumour samples, where patients were subjected to either radiotherapy or chemo-radiotherapy (Temozolomide-chemo), showed that surviving tumour cells were enriched in SUMO2 isoform; hence SUMO2 must be directly or indirectly involved in enabling GBM cells survival and resistance against Temozolomide chemo-radio therapeutic regimes. Probe 1 and Probe 2 refers to distinct cDNA probes used in microarray studies. **E** Kaplan Meier Survival plot from astrocytoma/GBM dataset (GSE-4271-GPL96) showed a significant association of high SUMO2 expression with reduced patient survival vs highly homologous isoform SUMO3. *p* value was derived from the log-rank test. High expression is indicated in red and low expression is indicated by blue-coloured curves. The numbers of patients in each group are indicated in the figure panel. **F** Top sub-panel: Microarray data (GEO-GSE72217) analysis of glioblastoma patients' tumour-derived cells from Human Glioma Cell Culture Repository, HGCC, showed significantly high expression of SUMO2 isoform vs other SUMO isoforms, *Middle sub panel:* RNA Seq data analysis (SRA-PRJNA508446) of glioblastoma tumour cells that were freshly isolated from glioblastoma patients' tumour tissues (primary cells), also showed significant expression of SUMO2 isoform, *Bottom sub-panel:* RNA Seq data (available at Cancer Cell Line Encyclopaedia, CCLE platform) showed that classically and often used glioblastoma cell lines had a significant expression of SUMO2 isoforms vs other isoforms. **G** RNA Seq data analysis from TCGA platform revealed that glioblastoma tissue sub-types (classical, mesenchymal, neural, proneural; based on the molecularly distinct transcriptome, hence plasticity) have significant expression of SUMO2 in all sub-type categories. Note that GBM molecular subtype signature information is used in clinical practice to determine GBM therapy's nature. **H** Microarray analysis on glioblastoma sub-types derived tumours cells (classical, mesenchymal, neural, proneural), from data available on HGCC platform confirms significant expression of SUMO2 in all GBM subtypes. All datasets are reported as mean ± SD. *p < 0.05, **p < 0.01, ***p < 0.001 and ****p < 0.0001; Mean is derived from a statistically significant number of samples, and Student t-test function was used to drive significance. For in-depth details on individual data points and dataset sample size in each graph, please refer to corresponding Additional tables mentioned in the main manuscript text

Analysis of RNA-Seq data by Gill et al. [37] [GSE59612] further showed that SUMO2 isoform was maximally upregulated in GBM core tissue and was also observed in tumour margins (Fig. 2A and Additional file 4: Table S22). Besides, pro-tumourigenic cancer stem cells (Fig. 2B, Additional file 4: Table S23), the tumour-promoting aberrant immune cells such as microglia and monocyte-derived macrophages [38], were also identified to express high levels of SUMO2 (Additional file 5: Figure S7 and Additional file 4: Table S24). Hence, SUMO2 was expressed throughout tumour heterogeneity.

Since the most aggressive GBMs arise from or contact the neural stem cell enriched lateral ventricular zones; analysis of the RNA-Seq data submitted by Mistry et al. [39] showed high expression of SUMO isoforms (except the SUMO4) in GBMs of both ventricular-sub ventricular neural stem cell regions (VSVZ +) and those that did not contact the ventricular stem cell linings (VSVZ-)(Fig. 2C and Additional file 4: Table S25).

SUMO2/3 high expression was also found to be predominantly associated with both chemo and radioresistant GBM samples, as per the analysis of microarray data submitted by Murat et al. [GSE7696] [40] (Fig. 2D and Additional file 4: Table S26), but amongst the two isoforms only SUMO2 was found to be significantly associated with the GBM patient reduced survival (Fig. 2E and Additional file 4: Table S27). This information suggests that identification of SUMO2 inhibitors and inhibition processes, which can target multiple GBM kinases/other upregulated pro-tumourigenic genes and render them non-functional or even degrade them, needs to be urgently initiated.

So at this juncture, we found it essential to consider whether a background toolbox is available for SUMO2 directed drug discovery against GBM. In order to facilitate the drug screening research, we found that both freshly derived patient GBM cell lines from HGCC (GEO-GSE72217, https://www.hgcc.se/, available for research) and other sources [Stringer et al., SRA-PRJNA508446] [41] as well as classically used GBM tumour cell lines (https://portals.broadinstitute.org/ccle), consistently upregulated transcriptome of SUMO2 (Fig. 2F and Additional file 4: Table S28), hence can be alternately used for anti-SUMO2 drug discovery.

The transcriptome of GBM patient tissue (TCGA) as well as patient derived cell lines (HGCC) under various molecularly distinct subtypes (classical, mesenchymal, neural and proneural) showed enhanced SUMO2 expression (Fig. 2G, H and Additional file 4: Table S29, 30).

### SUMO2 isoform is significantly expressed in both adult and pediatric GBMs and is essential for cancer cell fitness

**Fig. 3:**
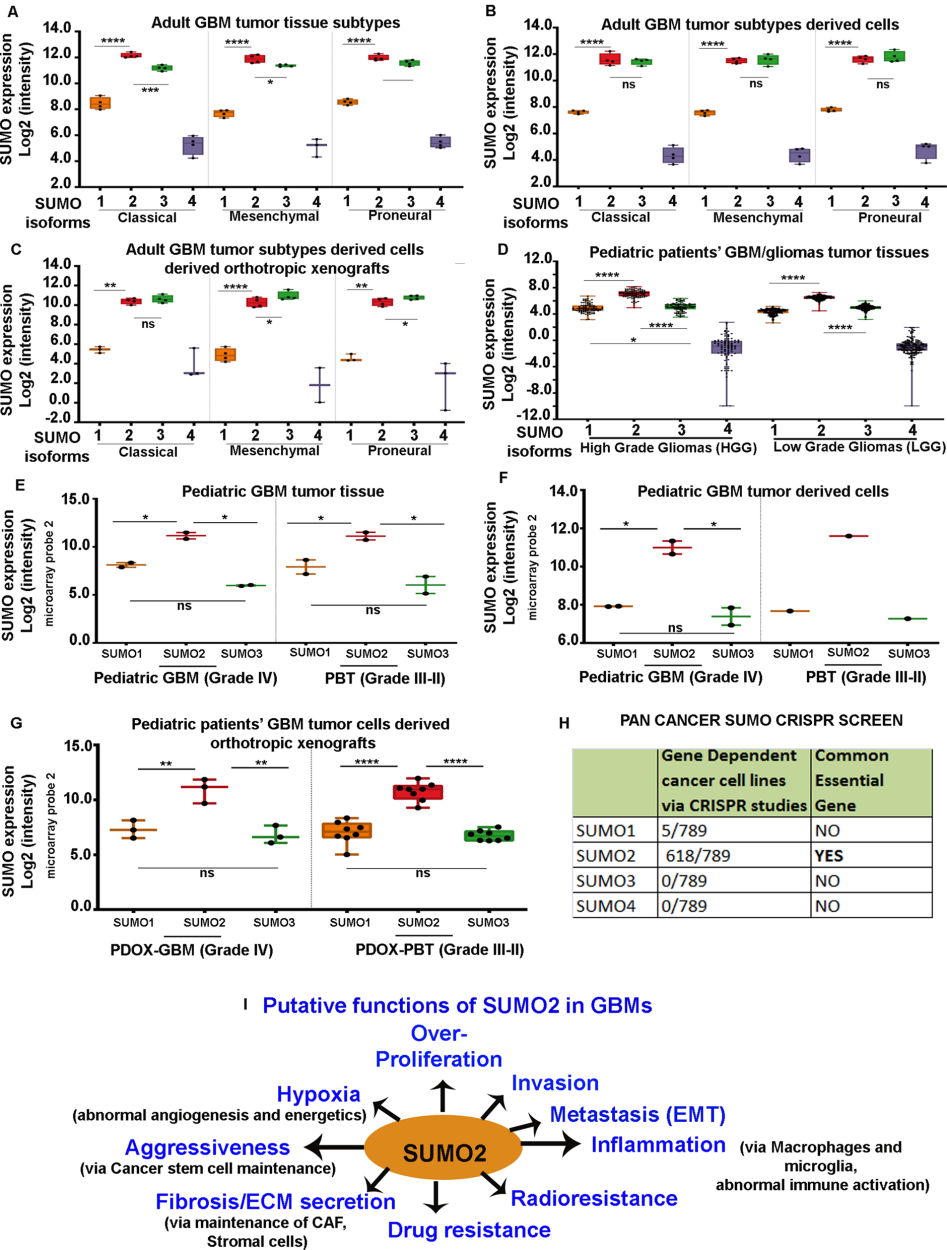
SUMO2 is a promising drug target for both adult and pediatric glioblastomas:** A, B, C** Microarray data from molecularly sub-typed (GEO-GSE118793) glioblastoma tumour tissues, the freshly isolated tumour cells from these sub-typed tumours and the orthotropic xenografts generated from the isolated tumours cells showed fidelity in high SUMO2 expression across classical, mesenchymal and proneural GBMs. **D** Pediatric Brain Atlas-CBTTC platform microarray data analysis from pediatric low and high-grade glioma tissues confirms high SUMO2 expression vs other SUMO isoforms. **E, F, G** Microarray data from pediatric glioblastoma (GBM, astrocytoma Grade IV; GEO-GSE99961) and astrocytomas (Grade II–III) tumour tissues, the freshly isolated tumour cells from these pediatric tumour grades, and the orthotropic xenografts generated from the isolated tumours cells showed fidelity in high SUMO2 expression. PDOX refers to Pediatric Orthotropic Xenografts; PBT refers to Pediatric Brian Tumours or astrocytomas between grades III-II. GBM is Grade IV astrocytoma. **H** CRISPR/CAS gene knockout based pan-cancer dependency test for SUMO2 in cancer cell survival fitness was extracted from DepMAP portal (https://depmap.org/portal/). The values in the table shows that 789 cancer cell lines were included in the study, wherein only SUMO2 isoform showed up as a common essential gene for the overall survival of cancer cells. **I** The schematic representation sums up the findings in this study which suggest that SUMO2 isoform by itself may be a key player in GBM development and progression via exercising its control on the genes involved in the hallmarks of cancer. Hence, SUMO2 is a promising target for anti-GBM therapeutics in all GBM subtypes. All datasets are reported as mean ± SD. *p < 0.05, **p < 0.01, ***p < 0.001 and ****p < 0.0001; Mean is derived from a statistically significant number of samples except in panels E, F, G and Student t-test function was used to drive significance. For in-depth details on individual data points and dataset sample size in each graph, please refer to corresponding additional tables mentioned in the main manuscript text

It is also worth noting that microarray-based transcriptomics analysis of GBM subtype tumours, freshly derived tumour cells from these cells and the respective orthotropic xenografts obtained from the freshly derived tumour cells, all showed similar high expression of SUMO2 [Stringer et al., GEO-GSE118793] [41](Fig. 3A–C and Additional file 4: Table S31).

Although pediatric GBM landscape is considered to be very different from the adult GBMs and the research is focused on finding different drugs for the two GBM types, we found that both pediatric low and high-grade gliomas expressed high levels of SUMO2 (Pediatric Brain Tumour Atlas: CBTTC from UCSC Xena) (Fig. 3D and Additional file 4: Table S32). Moreover, this was consistent with observed profiles in the adult primary and recurrent low and high gliomas (TCGA and CBTTC) (Additional file 5: Figure S8 and Additional file 4: Table S33). Besides, freshly derived cells from pediatric GBM tumours and orthotropic xenografts generated from them also showed consistently high SUMO2 expression [Fig. 3 E–G, cell lines are available for research; Additional file 4: Table S34] [42].

SUMOylation of cell cycle promoting EGFR kinase and SUMO2/3 involvement in astrocytic tumours have been documented; however, these were never considered or escalated seriously on translational platforms to become a part of GBM clinical management practice[12, 43].

There are some FDA-approved drugs (kerriamycin B, Spectinomycin B, topotecan) and plant-derived compounds (ginkgolic acid, tannic acid, anacardic acid) that target the SUMOylation process in general. Several other small molecule inhibitors are under development (GSK145A, 2- D08; SUMO deconjugating SENP1 inhibitors such as triptolide, momordine, compound J5, compound 4, compound 3, and compound 13 m) [10]. The use of these compounds in the clinical management of GBM has not materialized as general inhibition of SUMOylation over an extended period of administration can have several overt effects on the physiology and functioning of other organs [11].

GBMs are evidenced to recapitulate developmental processes, and indeed, even though SUMO-2 and SUMO-3 display ~ 95% sequence homology, SUMO-2 expression was significantly higher than SUMO-3 during development as in GBMs [Additional file 5: Figure S9A] [11]. Correlatively, it is also well documented that SUMO-2 knockout mice are embryonically lethal while SUMO-3 knockout mice are phenotypically normal [11].

As far as SUMO1 isoform is concerned, it has ~ 50% homology with SUMO-2/3 but has a lower expression than SUMO2 in developing mouse and human brain (Additional file 5: Figure S9A, Additional file 5: Figure S9B and Additional file 4: Table S35). Developmentally, SUMO2/3 can compensate for the loss of SUMO1; hence SUMO1 knockout mice are also viable[11]. This suggests that even in GBMs, where the microenvironment recapitulates early brain development, SUMO1 may be overall less significant than SUMO2.

Indeed, although both SUMO1 and SUMO2 have been identified to be expressed in glioma stem cells (Fig. 2B) [44, 45], and since human glioblastoma cells reflect a return to a foetal/embryonic developmental cell state [46], SUMO2 being indispensable to the development but not in adult physiology is plausibly a more potential target in anti-GBM therapeutics than SUMO1 in targeting glioblastoma stem cells.

In glioma/glioblastomas, even at protein levels, SUMO2 is found to be significantly highly expressed than SUMO1 (Additional file 5: Figure S6). This further indicates that SUMO2 alone targeting via intelligent structural design or by better understanding its transcriptional process, or even exploring the possibility of genetic manipulation of the SUMO2 translation pathway via microRNAs must be explored. There is also a possibility that even with very high sequence homology of SUMO2 with SUMO3, their conformational folding and transcriptional stimuli may vary, assigning them under distinct spatio-temporal regulatory controls, which may further attribute these isoforms with different functional roles.

SUMO2 has also been confirmed to be majorly upregulated in various other cancer cell lines (Additional file 5: Figure S10). Interestingly, CRISPR/CAS9 high throughput screening databases (Fig. 3H, www.depMap.org) that allow gene-specific cancer cell fitness assessment have shown SUMO2 to be a pan-cancer fitness gene vs other isoforms. Hence, the use of SUMO2 inhibition strategies merits investigation in both high and low-grade astrocytomas/gliomas. In addition, SUMO2 may be a crucial drug target in other tumours too, as indicated by patients' survival plots (Additional file 5: Figure S11, S12, Additional file 4: Table S36).

## Discussion:

We took proteo-transcriptomics approach to identify unique and promising drug target in GBM pathology and uncovered that (1) Key upregulated hub kinases and coding genes in GBM pathology controlling over-proliferation, hypoxia, stemness, ECM stiffening, inflammation, EMT, invasion, immune escape, chemo and radioresistance, are putative targets of SUMO2 conjugation, (2) SUMO2 is significantly expressed in adult primary and recurrent GBMs as well as in pediatric GBM tumours, (3) Orthotropic xenografts from adult and pediatric GBMs confirm high expression of SUMO2 in GBM tumour samples, (4) SUMO2 is significantly associated with patient survival plot and pan-cancer cell fitness, (5) Inhibition of SUMO2 isoform can potentially impair multiple oncogenic kinases as well as other key protumourigenic genes across GBM subtypes, in adult and pediatric patients alike, therefore, (6) Rationale design of SUMO2 inhibitors or search for its transcriptional inhibitors is urgently required for anti-GBM and potentially pan-cancer therapeutics.

Although we had set out on our exploration to target multiple pro-proliferation associated GBM kinases via inhibition of SUMOylation, we were intrigued to find that all key upregulated coding genes, involved in the generation and maintenance of the hallmarks of aggressive cancer, were SUMOylated. These observations jives with the fact that other than kinases; transcription factors (e.g. MITF, Myc, TFAP2A, FOXM1, FOXA2, RUNXs, HIF-1α, C/EBPbeta1, Ikaros, pRB, NDRG1, PCNA, IRF-1, NF-κB, ZEB1, p53, GATA3, VHL, SIRT1, BRCA1), signalling molecules (e.g. IQGAP1, Slug, RanGAP1,Rac1, MMPs), deubiquitinating enzyme (e.g. CYLD, Pontin, HDAC3), receptor proteins (e.g. TGFbR1, androgen receptor, estrogen receptors, progesterone receptor, glucocorticoid receptor, peroxisome proliferator activated receptor, retinoic acid receptor), DDR proteins (e.g. PML, DAXX, PRC1, MDC1, HERC2, RNF168, MRN complex, the homologous recombination proteins- Mre11, Rad50, Rad52 and Rad59, and the DNA damage checkpoint proteins Rad9 and Mrc1, Rad51, ATM, CHK1, DNA-PK and KU70/KU80), metabolic enzymes (e.g. Fatty-acid synthase, HMGS-1, PKM2), cell cycle regulators (e.g. CENP-A, CENP-E, BubR1, condensins, BLM helicase, and cohesin, Cyclin-E, Topoisomerase IIa, CHK1, Ki-67 and P53) are known substrates of SUMO isoforms [10, 47–55].

The novelty in our findings is that a significant proportion of the upregulated genes in GBM, involved in tumourigenesis, are found to be the targets of SUMO2/3 isoforms. However, since high expression of SUMO2 alone was found to be significantly associated with poor patient survival, this SUMO isoform could putatively regulate all GBM protumourigenic processes. The cancer community had almost lost hopes that such a ‘one size fits all’ kind of tumour target therapy may be possible with an exception to the CAR-T therapy (https://www.news-medical.net/news/20200214/Universal-One-Size-Fits-All-Cancer-Treatment.aspx). This is mainly due to the challenges posed by the heterogeneity of clinical and molecular data comprising of differences in age, gender, ethnicities, co-morbidities and mutations. In the context of GBMs, the primary tumours were held to be of a different molecular landscape than recurrent ones as the latter is evidenced to arise from more cancer stem cell-like populations [56, 57]. Therefore, a notion is held that primary and recurrent tumours will require different therapeutic regimes. Besides, the pediatric tumours are genetically and epigenetically identified to be different from that adult GBMs, again conforming to the opinion that exclusive treatment protocols needs to be developed for childhood GBMs [58]. Our analysis suggests that SUMO2 therapeutic inhibition will be beneficial to GBM patients irrespective of age, tissue differentiation and the type (primary or recurrent).

SUMOylation of proteins is clearly evidenced in cellular stress such as hypoxia, wherein a positive correlation is derived between SUMOylation and cancer growth, angiogenesis, glucose metabolism and stemness. Besides, SUMOylation of ATR and NFκB is identified as a critical component in DNA damage repair that enables resistance to chemo and radiotherapy, respectively [10]. DDX39B, a DExD/H-box RNA helicase involved in the downregulation of factors associated with the extracellular matrix, cellular migration, and angiogenesis, is degraded upon SUMOylation which in turn promotes resistance to alkylating chemotherapy in GBM [59]. Hence, several researches have demonstrated that pharmacological inhibition of the SUMO pathway may be a practical approach in overcoming cancer cell resistance to treatments. However, general inhibition of SUMOylation can be highly deleterious to health as it is a cytoprotective process [60, 61]. Our results indicate that mere suppression of SUMO2 isoform may be sufficient in drug sensitization vs global SUMOylation targeting.

Glioblastoma tumour-initiating cells are identified to be major culprits in GBM aggressiveness and relapse [62]. SUMOylation of Promyelocytic leukaemia protein (PML) facilitates its interaction with c-Myc, stabilising the protumourigenic c-Myc in glioma stem cells [10]. SUMOylation modification is reported in different stemness marker proteins like Oct-4, Oct-1, Nestin etc. [63–66]. Also, SUMOylation of retinoid acid receptors in stem cells induces resistance to RA-mediated cancer stem cells differentiation [67]. Since SUMO2 expression is found to be high in glioblastoma tumour-initiating stem cells, SUMO2 targeting is expected to greatly benefit in controlling tumour recurrence.

SUMO modified proteins such as Transducin β-like protein (TBL1) and Transducin β-like 1X-linked receptor 1 (TBLR1, coactivator for NF-κB-mediated transcription) are associated with inflammation-mediated promotion of tumourigenesis [10]. SUMOylation also contributes to the negative regulation of NKG2D and DNAM-1, reducing NK cell-mediated surveillance against tumours [68]. Since inflammation-driven immune escape and fibrosis (ECM stiffening) are key processes that drive glioblastoma pathogenesis, the high expression of SUMO2 in inflammation-promoting macrophages and stromal cells indicates that it may be crucial in their maintenance [69, 70]. Therefore, SUMO2 targeting may be helpful in reducing the numbers of such aberrant immune cells, which will invariably subside inflammatory secretions and cytokine storms.

Metastasis, migration and invasion associated cancer genes such as RanGAP1 (Ran-GTPase activating enzyme 1), Rho-like GTPase- Rac1, MMPs like MMP-9, MMP-14 and slug [10, 71] are found to be profoundly SUMOylated. Although metastasis is rare outside the central nervous system in GBMs, we find that SUMO2 is crucially associated with EMT genes, putatively supporting migration and invasion processes.

One of the major reasons for the extremely poor prognosis of GBMs is its asymptomatic nature in early stages, routine exosome profiling from blood serum can enable early diagnosis and thereby early staging of GBMs or precancerous lesions. Since our analysis suggests that SUMO2 specific conjugation of certain proteins putatively occurs in GBM, pull-down assays in combination with mass spectrometry may enable the identification of such SUMOylated tumour-specific biomarkers in exosomes. Given the reports that SUMO2 and SUMO2 conjugated proteins are identified in exosomes [72], SUMO2 and its target proteins can serve as a circulating biomarkers for determining cancer risk and for evaluating the chemotherapy response.

Further, most of the currently available anti-cancer drugs are weakly basic in nature, and the predominant acidic microenvironment of GBMs induces protonation of these drugs, inhibiting their entry into the tumour cells and the sites of action [73, 74]. Therefore, while using HTS library screening technologies to identify inhibitors of SUMO2 or its transcriptional inhibitors, due consideration should be laid on its cellular accessibility in the low pH GBM microenvironment. Additionally, combining SUMO2 inhibitors with FDA approved drugs and other anti-cancer phyto-compounds may efficiently target GBMs through drug synergism and may combat treatment resistance.

## Conclusions

We found that SUMO2 isoform was significantly over-expressed in both primary and recurrent GBM tumour tissues. Cellularly, both bulk and glioblastoma cancer stem cell-like population and as well as pro-tumourigenic macrophages/stromal cells over-expressed SUMO2 isoform. Molecularly, all major kinases and other genes involved in GBM formation and maintenance were found to be targets of SUMO2 conjugation. Clinically, SUMO2 isoform over-expression was significantly correlated with the shorter survival rates in the GBM patients. The over-expression was found in the GBMs of both adult and pediatric origins and also in the low-grade gliomas. In summary, SUMO2 is identified as the master regulator of the processes involved in generating cancer hallmarks; hence it is a promising anti-GBM drug target (Fig. 3I). Thus, our study calls for an urgent industry-academia collaborations to develop and validate SUMO2 isoform-specific inhibition strategies, which can target multiple hub pro-tumorigenic genes in glioblastoma pathology, irrespective of patient age and other clinicopathological parameters.

## Supplementary Information

**Additional file 1: Table S1.** GDC TCGA GBM clinical data of 173 samples.

**Additional file 2: Table S1.** GDC TCGA GBM mRNA expression normalized count data of 173 samples.

**Additional file 3: Table S1.** mRNA raw count data of 173 samples clinical samples associated with GDC TCGA GBM study.

**Additional file 4: Table S1–S36.** Tables associated with data shown in main and additional figures.

**Additional file 5: Methods and figures.** Materials and methods, addiitonal figures and legends.

## Data Availability

All data is available in the manuscript and its Additional material files.

## Abbreviations

AMPK: AMP-activated protein kinase
ATM: Ataxia-telangiectasia mutated serine/threonine kinase
ATR: Ataxia telangiectasia and Rad3-related protein serine/threonine kinase
BUB1B: Budding Uninhibited By Benzimidazoles 1 (Yeast Homolog) Beta- Mitotic Checkpoint Serine/Threonine Kinase
BRCA1: BReast CAncer gene 1
CAR-T: Chimeric antigen receptor T cells
CBTTC: Children's Brain Tumour Tissue Consortium
CCLE: Cancer Cell Line Encyclopedia
CDK: Cyclin-dependent kinase
CENP-A: Centromere protein A
CHK1: Checkpoint kinase 1
CPTAC: Clinical Proteomic Tumour Analysis Consortium
CYLD: CYLD lysine 63 deubiquitinase
DAVID: Database for Annotation, Visualization and Integrated Discovery
DAXX: Death Domain Associated Protein
DDR: DNA Damage Response
DNA-PK: DNA-dependent protein kinase
DNAM-1: DNAX Accessory Molecule-1
ECM: Extracellular matrix
EGFR: Epidermal growth factor receptor
EMT: Epithelial-to-Mesenchymal Transition
ERK5: Extracellular signal-regulated kinase 5
FC: Fold Change
FDA: Food and Drug Administration
FDR: False discovery rate
FGFR: Fibroblast growth factor receptors
FOXM1: Forkhead Box M1
FPKM: Fragments Per Kilobase of transcript per Million mapped reads
GBM: Glioblastoma multiforme
GDC: Genomic Data Commons Data Portal
GO: Gene ontology
GSE: Gene Expression Omnibus
GSEA: Gene Set Enrichment Analysis
GSK3: Glycogen synthase kinase 3
GTex: Genotype Tissue Expression portal
HGCC: Human Glioblastoma Cell Culture
HDAC3: Histone Deacetylase 3
HIF: Hypoxia Inducible Factor
HERC2: HECT and RLD domain containing E3 ubiquitin protein ligase 2
HMGS-1: 3-Hydroxy-3-Methylglutaryl-Coenzyme A Synthase HNF1A: Hepatocyte nuclear factor 1-alpha
HOX: Homeobox gene
HPA: Human Protein Atlas
IQGAP1: IQ Motif Containing GTPase Activating Protein 1
IRF-1: Interferon Regulatory Factor 1
IGFR: Insulin like growth factor 1
KRT19: Keratin 19
Lats1: Large tumour suppressor kinase 1
LIMK: LIM Domain Kinase 1
MAPK: Mitogen-activated protein kinase
MLL: Mixed Lineage Leukemia
MITF: Melanocyte inducing transcription factor
MNC: Maximum neighborhood component
MCC: Maximal clique centrality
MDC1: Mediator of DNA damage checkpoint 1
MMP: Matrix metalloproteinases
MPS1: Monopolar spindle 1
mTOR: Mammalian target of rapamycin
MRN: Mre11, Rad50 and Nbn gene complex
NDRG1: N-Myc Downstream Regulated 1
NPM1: Nucleophosmin
NK: Natural killer cells
NKG2D: Natural killer group 2D
NucleophosminNPM-ALK: Nucleophosmin-anaplastic lymphoma kinase
PAK: Serine/threonine p21-activating kinases
PBT: Pediatric Brain Tumour
PDC: Protein Data Commons
PDGFR: Platelet-derived growth factor receptor alpha
PDOX: Pediatric tumour derived orthotropic xenografts
PCNA: Proliferating cell nuclear antigen
PI3K: Phosphatidylinositol 3-kinase
PKD: Polycystic kidney disease
PKM2: Pyruvate kinase isozymes M1
PML: Promyelocytic leukemia protein
PRC1: Protein Regulator of cytokinesis 1
PTEN: Phosphatase and tensin homolog
RA: Retinoic acid
RhoA: Ras homolog gene family, member A
RNF168: Ring Finger Protein 168
ROCK: Rho-associated protein kinase
RSEM: RNA-seq by expectation–maximization
RUNX: Runt-related transcription factor
SENP1: Sestrin protease 1
SIRT1: Silent mating type information regulation 2 homolog 1
SUMO: Small ubiquitin-like modifier
TCGA: The Cancer Genome Atlas
TGFβR: Transforming growth factor beta receptor
TPM: Transcripts per million
TFAP2A: Transcription Factor AP-2 Alpha
UCSC Xena: University of California Santa Cruz, data browser
VEGFR: Vascular endothelial growth factor receptor
VHL: Von Hippel–Lindau syndrome
ZEB1: Zinc Finger E-Box Binding Homeobox 1
