# Supplementary material for: Proteo-transcriptomics meta-analysis identifies SUMO2 as a promising target in glioblastoma multiforme therapeutics
Source: Cancer Cell Int. 2021 Oct 29;21:575. doi: 10.1186/s12935-021-02279-y (PMC8555349; doi:10.1186/s12935-021-02279-y)
Supplement: Supplementary file 5 — Additional file 5: Methods and figures. Materials and methods, addiitonal figures and legends. [file 12935_2021_2279_MOESM5_ESM.pdf]

Additional File 5

Supplementary Materials For

'Proteo-Transcriptomics meta-analysis identifies SUMO2 as a promising target in glioblastoma multiforme therapeutics'

Aswani Krishna P, Sebastian John, Puja LaxmanRao Shinde and Rashmi Mishra

*Correspondence to:rashmimishra@rgcb.res.in;rashmi.mpi.cbg@gmail.com

This file includes:

I) Supplementary Methods

II) Supplementary Figures Figs.S1-S12 with Figure Legends

Materials and Methodology

1) R code for converting log2(count+1) data to raw htseq counts and obtaining differentially expressed genes using DESeq2

```
#converting log2(Count+1) data to raw count

TCGA.GBM.htseq_counts <- read.delim("D:/TCGA/data/DATA/TCGA-GBM.htseq_counts.tsv",
row.names=1)

x <- data.matrix(TCGA.GBM.htseq_counts)

x[,1:173]<- as.integer(((2^x[,1:173])-1))

write.table(x,file="D:/TCGA/data/DATA/gbm_htseq_counts.tsv")

#Differentially Expressed gene analysis

library(DESeq2)

gbm.counts` <- read.csv("D:/TCGA/gbm_htseq_counts.tsv", row.names=1)

  View(gbm_htseq_counts.tsv)

phenotype <- read.csv("D:/TCGA/phenotype.csv", row.names=1)

View(phenotype)

cts<-as.matrix(`gbm_htseq_counts.tsv`)

coldata<-phenotype

all(rownames(coldata) %in% colnames(cts))

[1] TRUE

all(rownames(coldata) == colnames(cts))

[1] FALSE

cts <- cts[, rownames(coldata)]

all(rownames(coldata) == colnames(cts))

[1] TRUE


library("DESeq2")

dds <- DESeqDataSetFromMatrix(countData = cts,

                              colData = coldata,

                              design = ~ condition)

dds

keep <- rowSums(counts(dds)) >= 10

dds <- dds[keep,]

dds$condition <- factor(dds$condition, levels = c("control","tumor"))

dds <- DESeq(dds)

res <- results(dds)

res
```

2) Website links for various datasets used in this study along with citations.

SL. NO	DATA	SITE	Link/ID
1	TCGA GBM Level 3 data	UCSC XENA	https://xenabrowser.net/datapages/?dataset=TCGA-GBM.htseq_counts.tsv&host=https%3A%2F%2Fgdc.xenahubs.net&removeHub=https%3A%2F%2Fxena.treehouse.gi.ucsc.edu%3A443<br><br>**Citation:** Goldman, M.J., Craft, B., Hastie, M. et al. Visualizing and interpreting cancer genomics data via the Xena platform. Nat Biotechnol. (2020). https://doi.org/10.1038/s41587-020-0546-8
2	Survival analysis	PrognoScan	http://dna00.bio.kyutech.ac.jp/PrognoScan/<br><br>**Citation:** Mizuno, Hideaki,et al."PrognoScan: a new data base for meta-analysis of the prognostic value of genes." BMC medical genomics 2.1 (2009):18.
3	Normal vs Tumour RNASeq	UCSC XENA	https://xena.ucsc.edu/compare-tissue/<br><br>**Citation:** Goldman, M.J., Craft, B., Hastie, M. et al. Visualizing and interpreting cancer genomics data via the Xena platform. Nat Biotechnol. (2020). https://doi.org/10.1038/s41587-020-0546-8
4	Tumour Cell line information	CCLE	https://portals.broadinstitute.org/ccle<br><br>**Citation:** Ghandi, Mahmoud, et al. "Next-generation characterization of the cancer cell line encyclopedia." Nature 569.7757(2019):503-508.
5	MRI-Non-neoplastic, Margin, core data GBM data	GEO-GSE59612-RNA-Seq<br><br>PMID-25114226	https://www.ncbi.nlm.nih.gov/geo/query/acc.cgi?acc=GSE59612<br><br>**Citation:** Gill, BrianJ., et al."MRI-localized biopsies reveal subtype-specific differences in molecular and cellular composition at the margins of glioblastoma." Proceedings of the National Academy of Sciences 111.34(2014):12550-12555.
6	Radio/chemo GBM data	GEO-GSE7696-Microarray PMID-18565887, 21642372	https://www.ncbi.nlm.nih.gov/geo/query/acc.cgi?acc=GSE7696<br><br>**Citation**: Murat A, Migliavacca E,Gorlia T, Lambiv WL et al. Stem cell-related "self-renewal" signature and high epidermal growth factor receptor expression associated with resistance to concomitant chemo radiotherapy in glioblastoma.<br>J<br>Clin Oncol 2008 Jun 20;26(18):3015-24.PMID:18565887
7	GBM Subtype data	From literature and TCGA data	**Citation:** Verhaak, Roel GW, et al. "Integrated genomic analysis identifies clinically relevant subtypes of glioblastoma characterized by abnormalities in PDGFRA, IDH1, EGFR, and NF1." Cancer cell 17.1 (2010): 98-110.

8	SVZ DATA	From literature data and TCGA-samples	**Citation:** Mistry, Akshit kumar M., et al. “Ventricular-subventricular zone contact by glioblastoma is not associated with molecular signatures in bulk tumour data.” *Scientific reports* 9.1 (2019):1-12.
9	HGCC-CELL LINE DATA	https://www.hgcc.se/ GEO-GSE72217-Microarray PMID-26629530	https://www.ncbi.nlm.nih.gov/geo/query/acc.cgi?acc=GSE72219<br><br>**Citation:** Xie,Yuan,et al."The human glioblastoma cell culture resource: validated cell models representing all molecular subtypes." EBioMedicine 2.10 (2015):1351-1363.
10	GBM Cell line, tumour tissue, Xenograft	GEO-GSE118793-<br><br>Microarray SRA-PRJNA508446	https://www.ncbi.nlm.nih.gov/geo/query/acc.cgi?acc=GSE118793; https://www.ncbi.nlm.nih.gov/bioproject/PRJNA508446/<br><br>**Citation:** Stringer, Brett W., et al. "A reference collection of patient-derived cell line and xenograft models of proneural, classical and Mesenchymal glioblastoma."Scientific reports 9.1 (2019):1-14.
11	Pediatric data-HGG & LGG Adult-HGG &LGG	CBTTC-From UCSC Xena	https://xenabrowser.net/datapages/?cohort=Pediatric%20Brain%20Tumour%20Atlas%3A%20CBTTC&removeHub=https%3A%2F% 2Fxena.treehouse.gi.ucsc.edu%3A443
12	Tumour immune Microenvironment study for SUMO Isoforms expression	Braintime-MG vs MDM -Microarray	https://joycelab.shinyapps.io/braintime/<br><br>**Citation:** Klemm, Florian, et al. "Interrogation of the Microenvironmental Landscape in Brain Tumours Reveals Disease-Specific Alterations of Immune Cells." Cell (2020).
13	Sumo protein expression by Immunohistoc-hemistry	Human protein atlas-using R package-HPAanalyze	**Citation:** Tran, AnhNhat, et al.“HPAanalyze:an Rpackage that facilitates the retrieval and analysis of the Human Protein Atlas data.” BMC bioinformatics 20.1 (2019):463.
14	GBM-SUMO Isoforms expression in cancer stem cells	IVYGAP-RNASeq	https://glioblastoma.alleninstitute.org/; https://glioblastoma.alleninstitute.org/api/v2/well_known_file_download/305873915<br><br>**Citation:** Puchalski R.B. et al. (2018) An anatomic transcriptional atlas of human glioblastoma, Science 360(6389):660-663 doi:10.1126/science.aaf2666
15	Protein data commons (PDC)	PDC-Proteome data-Mass spectrometry	https://proteomic.datacommons.cancer.gov/pdc/https://proteomic.datacommons.cancer.gov/pdc/study/PDC000204
16	Sumo gene expression in developing human	Allen brain atlas-RNASeq	https://portal.brain-map.org/http://www.brainspan.org/static/download.html<br><br>**Citation:** Miller,J.A .et al.(2014)Transcriptional landscape of the prenatal human brain, Nature 508: 199-06.doi:10.1038/nature13185

17	Cluster profiler	Over representation analysis	**Citation:** Yu, Guangchuang, et al. "clusterProfiler: an R package for comparing biological themes among gene clusters." Omics: a journal of integrative biology 16.5 (2012):284-287.
18	Pediatric GBM Cell line data	GEO-GSE99961 Microarray Data	https://www.ncbi.nlm.nih.gov/geo/query/acc.cgi?acc=GSE99961<br><br>**Citation:** Brabetz S, Leary SES, Gröbner SN, Nakamoto MW et al. A biobank of patient-derived pediatric brain tumour models. Nat Med 2018 Nov;24(11):1752-1761.PMID: 30349086
19	GSEA	GSEA TOOL	https://www.gsea-msigdb.org/gsea/index.jsp<br><br>**Citation:** Subramanian, Aravind, et al. "Gene set enrichment analysis: a knowledge-based approach for interpreting genome-wide expression profiles." Proceedings of the National Academy of Sciences102.43(2005):15545-15550
20	CRISPR DATA	DEPMAP	**For DepMap release:**<br>DepMap, Broad (2020): DepMap 20Q4 Public. figshare. Dataset doi:10.6084/m9.figshare.13237076.v2.<br><br>**For CRISPR datasets:**<br>Robin M.Meyers, Jordan G.Bryan,James M.McFarland,Barbara A.Weir,David E.Root,William C.Hahn, Aviad Tsherniak.Computational correction of copy number effect improves specificity of CRISPR-Cas9 essentiality screens in cancer cells. Nature Genetics 2017 October 49:1779–1784.doi:10.1038/ng.3984<br><br>Dempster, J.M.,Rossen, J.,Kazachkova, M.,Pan,J., Kugener,G.,Root, D. E., & Tsherniak, A. (2019). Extracting Biological Insights from the Project Achilles Genome-Scale CRISPR Screens inCancer Cell Lines. BioRxiv,720243.
21	DAVID	Functional Enrichment Analysis	https://david.ncifcrf.gov/<br><br>**Citation:** Huang DW ,Sherman BT,Lempicki RA. Systematic and integrative analysis of large gene lists using DAVID bioinformatics resources. Nat Protoc. England; 2009;4:44–57.

SUPPLEMENTARY FIGURE LEGENDS:

Figure S1: Kinases expression in primary and recurrent glioblastoma multiforme (GBMs): Heatmap representation is shown to depict upregulated, unchanged and downregulated kinases in both primary and recurrent GBMs. Primary GBM fold change is calculated with respect to adjacent normal tissue control samples: Similarly, recurrent GBM fold change is calculated with respect to adjacent normal tissue control samples. Colour bar corresponds to per-gene log2 fold change values in compared groups.

Figure S2: Volcano plot representation of upregulated, unchanged and downregulated kinases expression in GBMs: (A-B) Volcano plot shows that several kinases expressions are changed with respect to adjacent normal tissues in both primary and recurrent GBMs. **(C)** The Volcano plot shows that only a few kinases are uniquely upregulated or downregulated in primary GBM vs recurrent GBM. Hence, most kinases expressions are coordinated similarly in primary and recurrent GBMs.

Figure S3: Pie chart representation of glioblastoma upregulated kinases and coding genes identified as targets of SUMO2/3: Analysis of Hendriks et al., 2018 mass spectrometry-based proteomics data revealed that a substantial percentage of GBM upregulated kinases and coding genes are targets of SUMO2/3 isoforms (represented as an orange colour fraction in the pie chart).

Figure S4: Over-representation test for extracting biological processes enrichment in primary GBM upregulated coding genes that are identified as targets of SUMO2/3: Dot plot showing top 50 representative enriched biological processes for upregulated coding genes in primary GBMs that are identified as targets of SUMO 2/3 via mass spectrometry data of Hendriks et. al. 2018. Each node represents the enrichment by node colour and circle, size, colour of the dot indicates the significance (padj value), and the size of the dot represents the number of genes associated with the enriched GO terms.

Figure S5: Over-representation test for extracting biological processes enrichment analysis in recurrent GBM upregulated coding genes that are identified as targets of SUMO2/3: Dot plot showing top 50 representative enriched biological processes for upregulated coding genes in recurrent GBM that are identified as targets of SUMO2/3 via mass spectrometry data of Hendriks et.al. 2018. Each node represents the enrichment by node colour and circle size, colour of the dot indicates the significance (padj value), and the size of the dot represents the number of genes associated with the enriched GO terms.

Figure S6: SUMO2 isoform shows enrichment in GBMs at protein levels also: A) Protein Data Commons extracted mass spectrometry data on 11 GBM samples shows higher expression of SUMO2 isoform over SUMO1 in the same dataset. All datasets are reported as mean±SD.*p<0.05,**p<0.01,***p<0.001and****p<0.0001; Mean is derived from a statistically significant number of samples, and Student t-Test function was used to drive significance. **B)** Information on SUMO isoform expression levels (high, medium, low) in immunostained patient tumour samples was extracted from in Human Protein Atlas dataset and plotted as a bar graph using hpa*VisPatho* R program code, created by Tran et al., 2019 (doi:10.1186/s12859-019-3059-z).**(C)** Representative images for expression of SUMO2 in normal brain regions(cerebral cortex) and glioma patient tissue samples shows higher expression in the pathology.

Figure S7: SUMO2 isoform is expressed in GBM protumourigenic immune cells: (A-D) RNASeq data analysis from Klemm et al. study (*Cell.* 2020; doi: 10.1016/j.cell.2020.05.007) identifies SUMO isoforms and SUMO2 significant expressions in glioma tumour-associated microglia (MG), monocytes derived macrophages (MDM) and brain metastatic cells (BrM). For in-depth details on individual data points and dataset sample size in each graph, please refer to corresponding supplementary tables mentioned in the main manuscript text.

Figure S8: SUMO2 isoform is highly expressed in both low-grade gliomas and high-grade GBMs of adult and pediatric origins: RNA Seq data analysis from pediatric glioma (CBTTC platform) and adult gliomas (TCGA portal for adult gliomas/GBM studies) respectively reveals pediatric GBM and low grade pediatric glioma tissues as well as adult primary and recurrent low grade gliomas/ GBMs have higher expression of SUMO2 isoform vs other SUMO isoforms and normal controls. For in-depth details on individual data points and datasets sample size in each graph, please refer to corresponding supplementary tables mentioned in the main manuscript text.

All datasets are reported as mean± SD. *p<0.05, **p<0.01, ***p<0.001 and ****p<0.0001; Mean is derived from a statistically significant number of samples, and Student t-Test function was used to drive significance.

Figure S9: SUMO2 isoforms shows abundance in developing human and mouse brains: **(A)** Developing human brain (8-37 pcw) RNASeq data from the Allen Brain Atlas shows significantly higher expression of SUMO2 vs other SUMO isoforms. For in-depth details on individual data points and dataset sample size in each graph, please refer to corresponding supplementary tables mentioned in the main manuscript text.

All datasets are reported as mean± SD. *p<0.05, **p<0.01, ***p<0.001 and ****p<0.0001; Mean is derived from a statistically significant number of samples, and Student t-Test function was used to drive significance.

(B) SUMO1 and SUMO2 transcriptome enrichment data in developing mouse brain (*via* in situ-hybridization and whole- mount mapping embryonic age 14.5 dpc,) showed SUMO2 to be more highly expressed in brain vs SUMO1.

Data images were extracted from EMAGE; which is an electronic mouse atlas; weblink:emouseatlas.org/emagewebapp/pages/emage_data_browse.jsf. The image source for SUMO1 is as follows-ID: EMAGE:16292;(http://www.emouseatlas.org/gxdb/dbImage/segment4/16292/detail_16292.html; http://www.emouseatlas.org/gxdb/dbImage/segment4/16292/16292_WM_1.png)

The image data source for SUMO2 is as follows: ID: EMAGE: 16940; (http://www.emouseatlas.org/gxdb/dbImage/segment4/16940/detail_16940.html; http://www.emouseatlas.org/gxdb/dbImage/segment4/16940/16940_WM_1.png)

Annotation colour keys for depicting expression levels are shown adjacent to the figure and are derived from the source files.

Figure S10: Cancer Cell Line Encyclopedia repository (CCLE) data shows SUMO2 isoform enrichment in several human cancer cell lines: (A-D) SUMO isoform mRNA expression levels in various cancer cell lines representing tumours of different organ origins was extracted and plotted. Numbers on x-axis of the graphs correspond to respective tumours as shown in the schema on the top of panel A. Data show high expression of SUMO2 in many other cancers besides gliomas/glioblastomas.

Figure S11: Survival plots show association of SUMO2 high expression with poor prognosis in breast cancers: (A-F) Kaplan Meier Survival plot (Distant Metastasis Free Survival) from various breast cancer datasets, mentioned in the respective figure panels, showed a significant association of high SUMO2 expression with reduced patient survival. *P* value was derived from the log-rank test. High expression is indicated in red and low expression is indicated by blue-coloured curves. Numbers of patients in each group are indicated in the figure panel.

Figure S12: Survival plots show association of SUMO2 high expression with poor prognosis in colorectal and lung cancers: (A-D) Kaplan Meier Survival plot (Overall Survival) from Colorectal and Lung cancer datasets, mentioned in the respective figure panels, showed a significant association of high SUMO2 expression with reduced patient survival. *P*-value was derived from the log-rank test. High expression is indicated in red and low expression is indicated by blue-coloured curves. The numbers of patients in each group are indicated in the figure panel.

1. Kinases expression in Primary GBM vs. Adjacent Normal Brain Tissue
2. Kinases expression in Recurrent GBM vs. Adjacent Normal Brain Tissue
3. Kinases expression in Primary GBM vs. Recurrent GBM

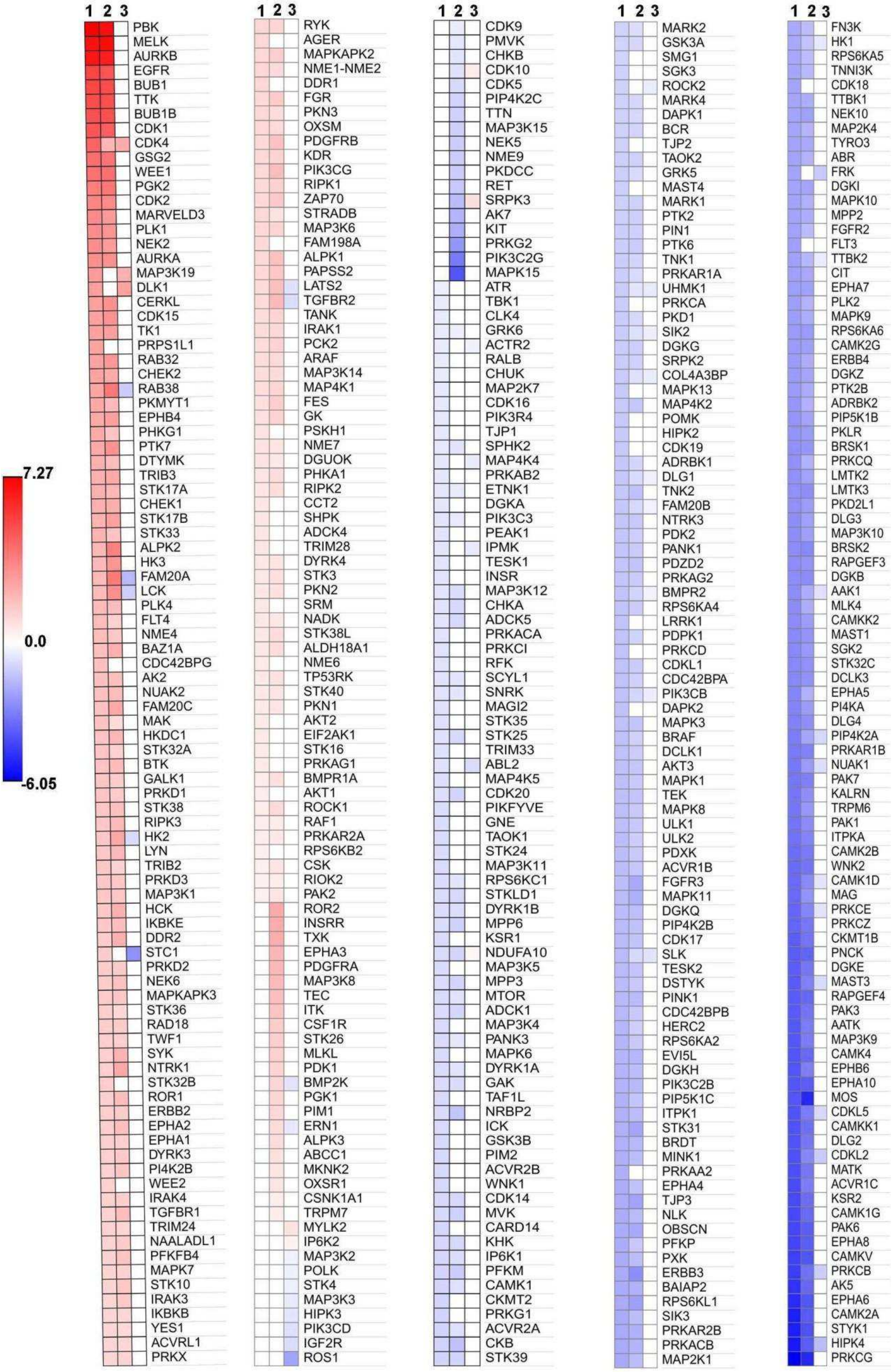

Figure S1

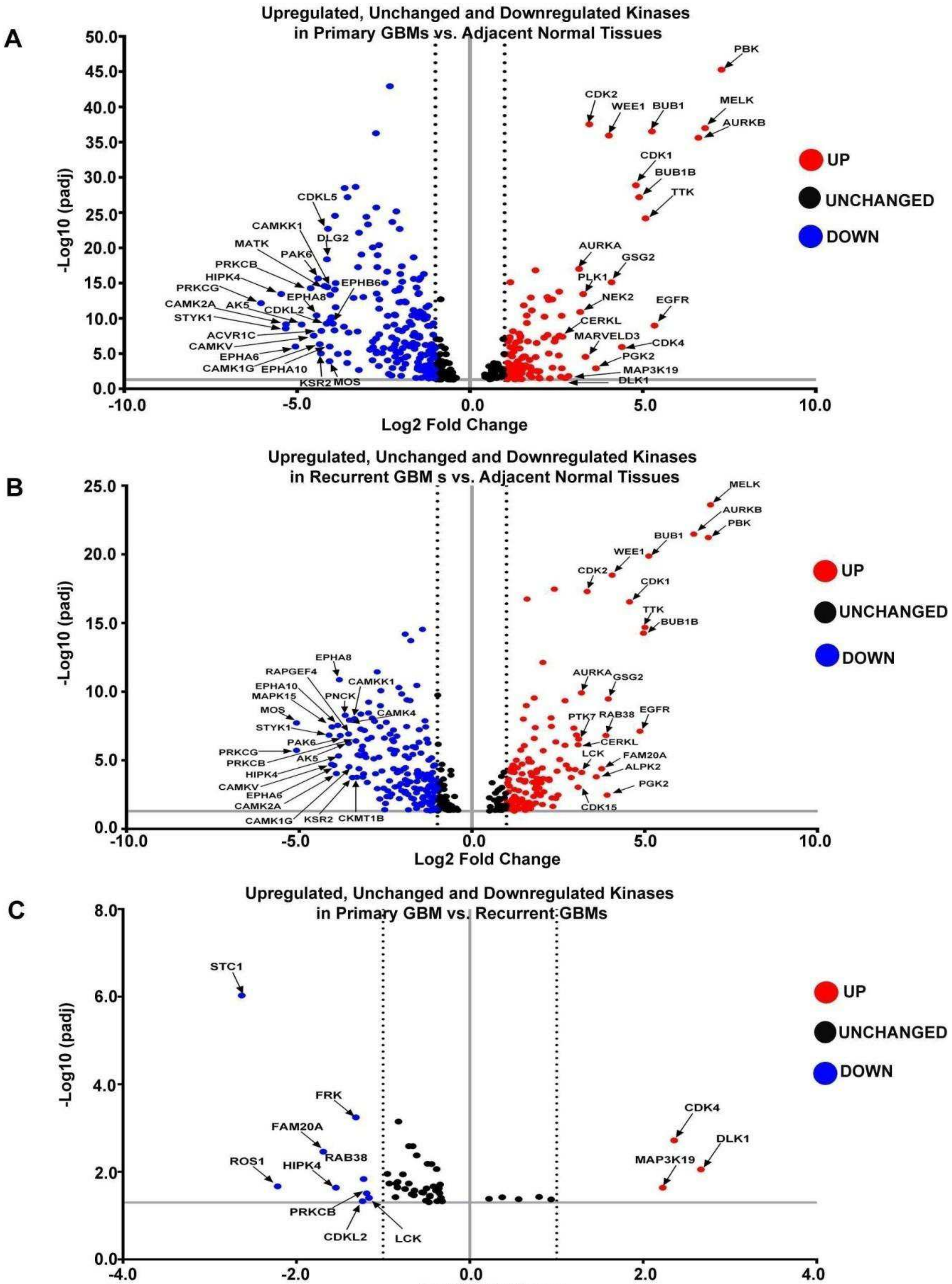

A
Upregulated, Unchanged and Downregulated Kinases
in Primary GBMs vs. Adjacent Normal Tissues
-Log10 (padj)
Log2 Fold Change
UP
UNCHANGED
DOWN
B
Upregulated, Unchanged and Downregulated Kinases
in Recurrent GBM s vs. Adjacent Normal Tissues
C
Upregulated, Unchanged and Downregulated Kinases
in Primary GBM vs. Recurrent GBMs

FIGURE S2

UPREGULATED Kinases in GBM
putatively modified by SUMO2/3

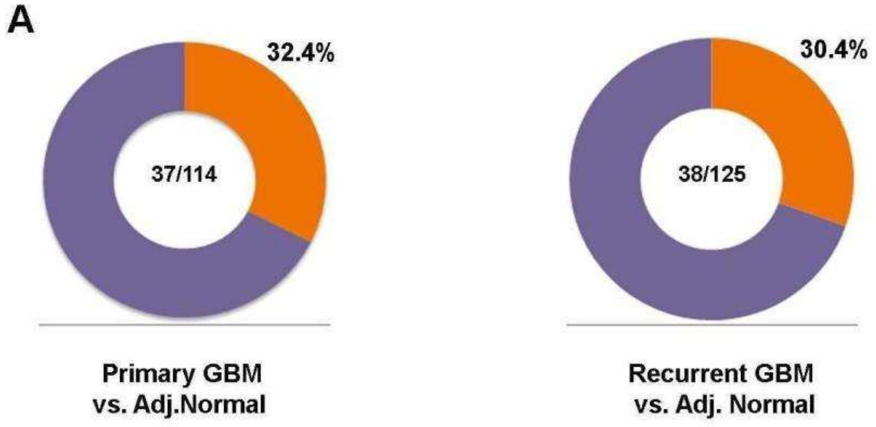

UPREGULATED Coding Genes in GBM
putatively modified by SUMO2/3

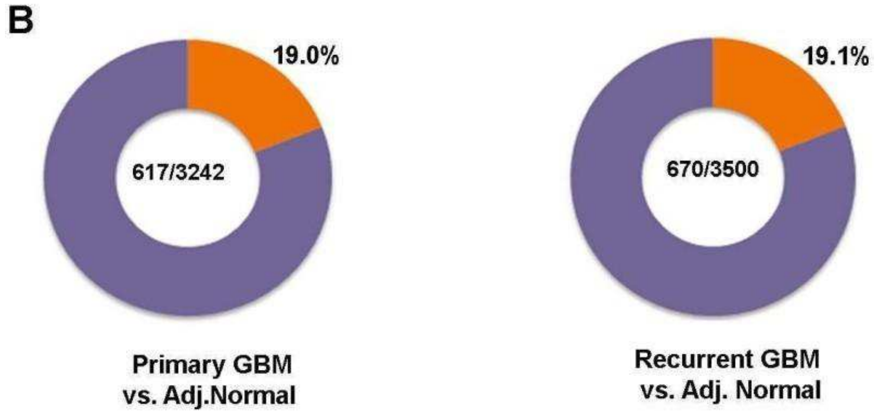

FIGURE S3

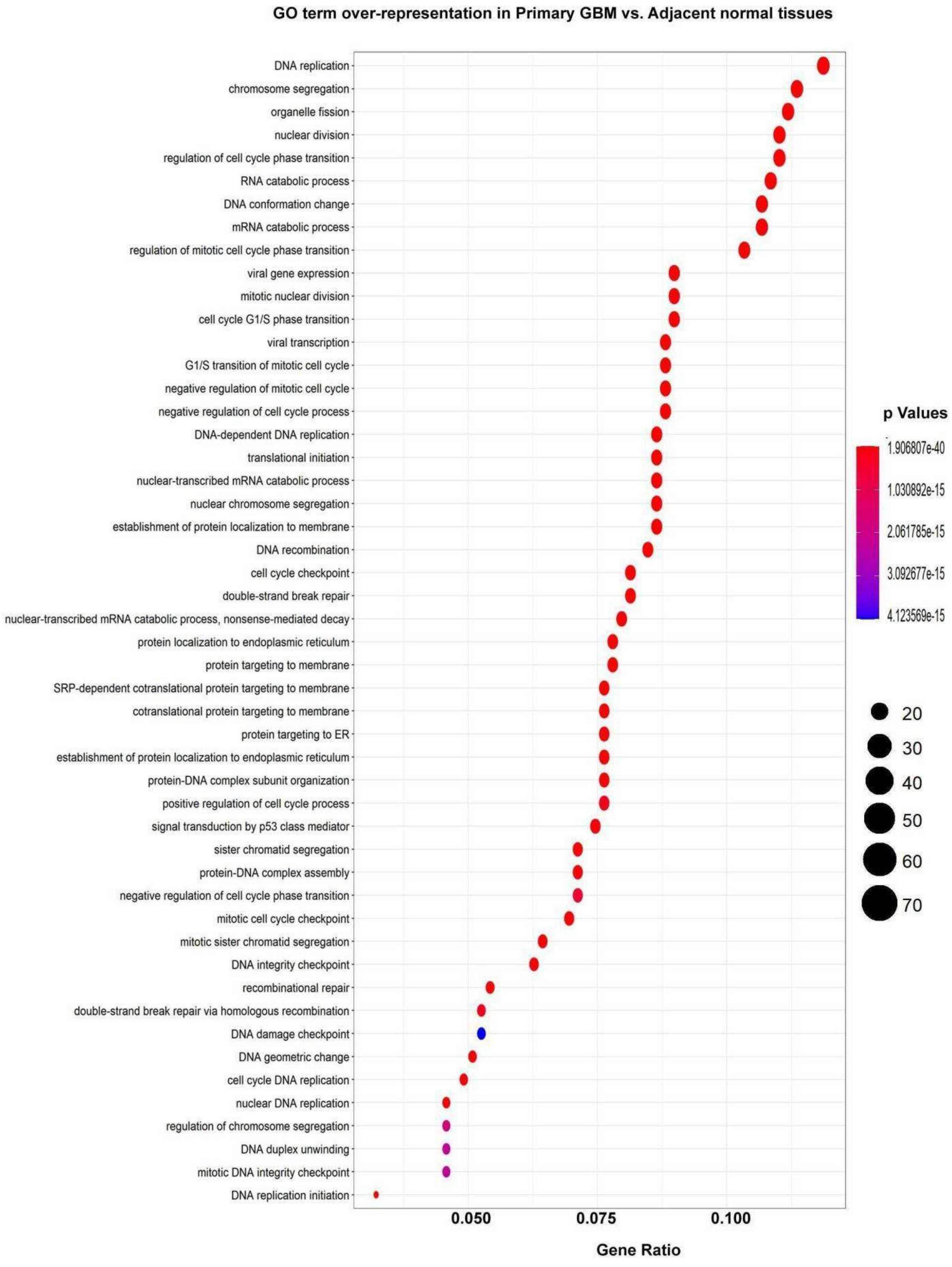
GO term over-representation in Primary GBM vs. Adjacent normal tissues
DNA replication
chromosome segregation
organelle fission
nuclear division
regulation of cell cycle phase transition
RNA catabolic process
DNA conformation change
mRNA catabolic process
regulation of mitotic cell cycle phase transition
viral gene expression
mitotic nuclear division
cell cycle G1/S phase transition
viral transcription
G1/S transition of mitotic cell cycle
negative regulation of mitotic cell cycle
negative regulation of cell cycle process
DNA-dependent DNA replication
translational initiation
nuclear-transcribed mRNA catabolic process
nuclear chromosome segregation
establishment of protein localization to membrane
DNA recombination
cell cycle checkpoint
double-strand break repair
nuclear-transcribed mRNA catabolic process, nonsense-mediated decay
protein localization to endoplasmic reticulum
protein targeting to membrane
SRP-dependent cotranslational protein targeting to membrane
cotranslational protein targeting to membrane
protein targeting to ER
establishment of protein localization to endoplasmic reticulum
protein-DNA complex subunit organization
positive regulation of cell cycle process
signal transduction by p53 class mediator
sister chromatid segregation
protein-DNA complex assembly
negative regulation of cell cycle phase transition
mitotic cell cycle checkpoint
mitotic sister chromatid segregation
DNA integrity checkpoint
recombinational repair
double-strand break repair via homologous recombination
DNA damage checkpoint
DNA geometric change
cell cycle DNA replication
nuclear DNA replication
regulation of chromosome segregation
DNA duplex unwinding
mitotic DNA integrity checkpoint
DNA replication initiation
0.050
0.075
0.100
Gene Ratio
p Values
1.906807e-40
1.030892e-15
2.061785e-15
3.092677e-15
4.123569e-15
20
30
40
50
60
70

Figure S4

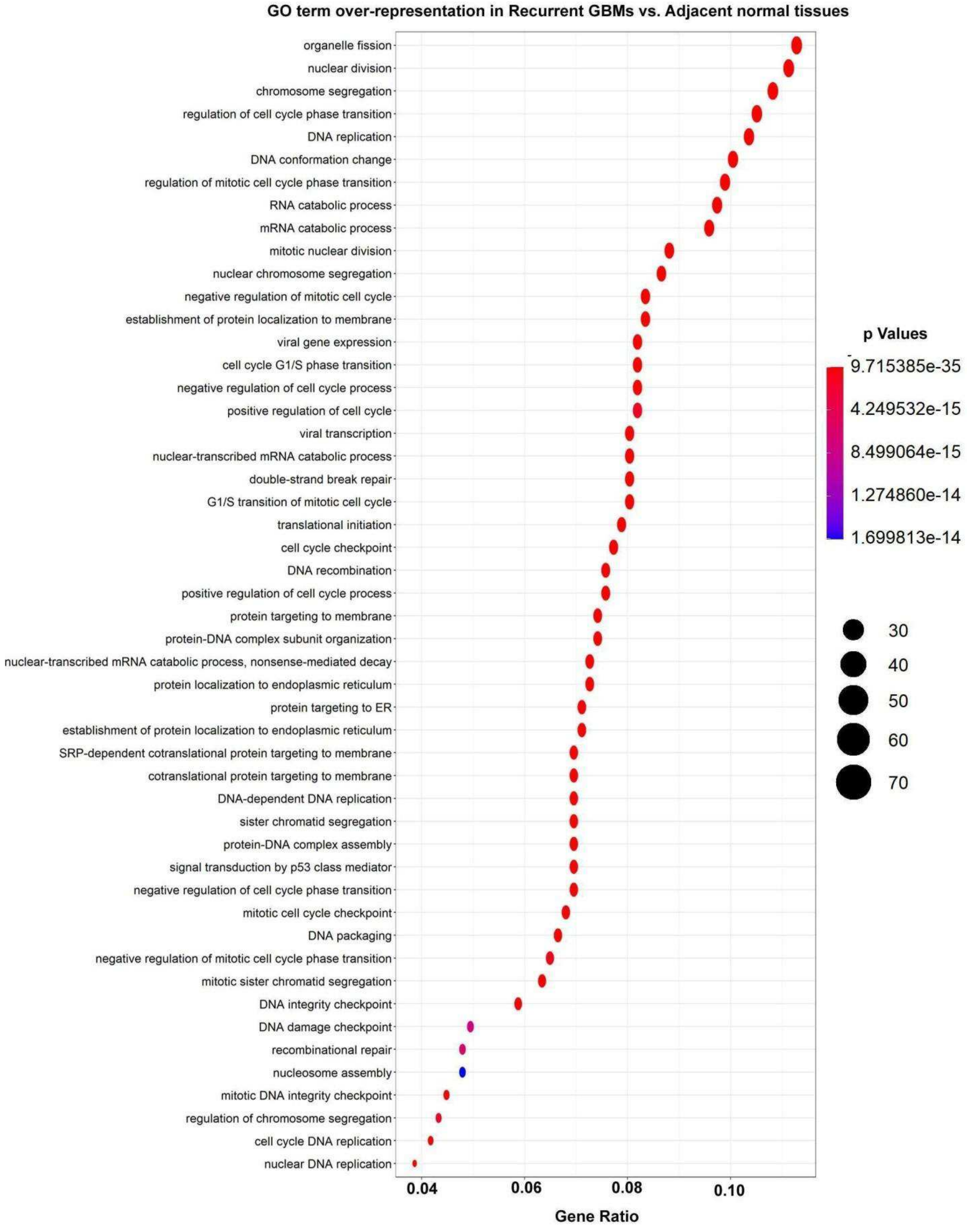
GO term over-representation in Recurrent GBMs vs. Adjacent normal tissues
organelle fission
nuclear division
chromosome segregation
regulation of cell cycle phase transition
DNA replication
DNA conformation change
regulation of mitotic cell cycle phase transition
RNA catabolic process
mRNA catabolic process
mitotic nuclear division
nuclear chromosome segregation
negative regulation of mitotic cell cycle
establishment of protein localization to membrane
viral gene expression
cell cycle G1/S phase transition
negative regulation of cell cycle process
positive regulation of cell cycle
viral transcription
nuclear-transcribed mRNA catabolic process
double-strand break repair
G1/S transition of mitotic cell cycle
translational initiation
cell cycle checkpoint
DNA recombination
positive regulation of cell cycle process
protein targeting to membrane
protein-DNA complex subunit organization
nuclear-transcribed mRNA catabolic process, nonsense-mediated decay
protein localization to endoplasmic reticulum
protein targeting to ER
establishment of protein localization to endoplasmic reticulum
SRP-dependent cotranslational protein targeting to membrane
cotranslational protein targeting to membrane
DNA-dependent DNA replication
sister chromatid segregation
protein-DNA complex assembly
signal transduction by p53 class mediator
negative regulation of cell cycle phase transition
mitotic cell cycle checkpoint
DNA packaging
negative regulation of mitotic cell cycle phase transition
mitotic sister chromatid segregation
DNA integrity checkpoint
DNA damage checkpoint
recombinational repair
nucleosome assembly
mitotic DNA integrity checkpoint
regulation of chromosome segregation
cell cycle DNA replication
nuclear DNA replication
0.04
0.06
0.08
0.10
Gene Ratio
p Values
9.715385e-35
4.249532e-15
8.499064e-15
1.274860e-14
1.699813e-14
30
40
50
60
70

Figure S5

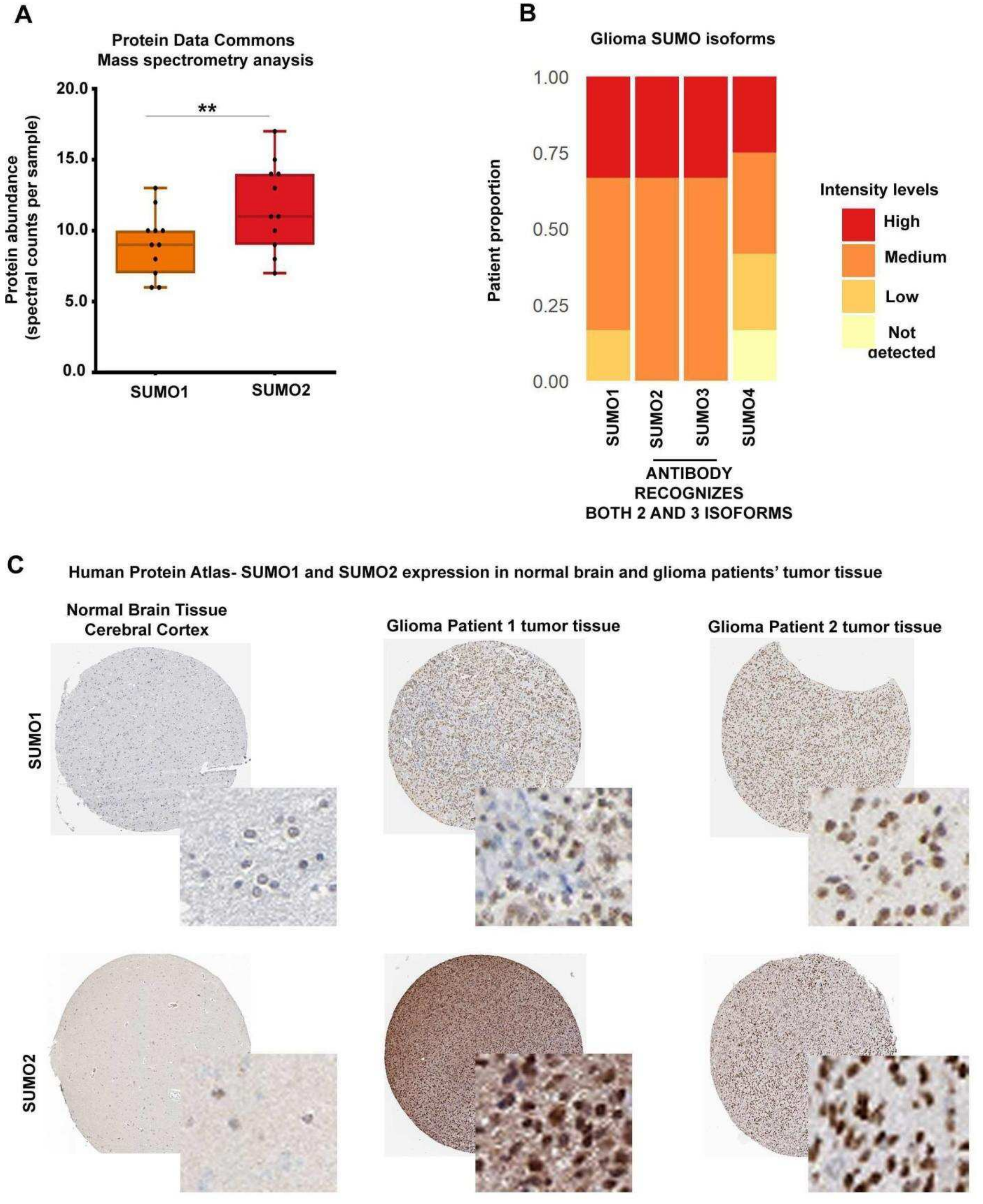
A
Protein Data Commons
Mass spectrometry anaysis
Protein abundance
(spectral counts per sample)
20.0
15.0
10.0
5.0
0.0
**
SUMO1
SUMO2
B
Glioma SUMO isoforms
Patient proportion
1.00
0.75
0.50
0.25
0.00
SUMO1
SUMO2
SUMO3
SUMO4
ANTIBODY
RECOGNIZES
BOTH 2 AND 3 ISOFORMS
Intensity levels
High
Medium
Low
Not
detected
C
Human Protein Atlas- SUMO1 and SUMO2 expression in normal brain and glioma patients' tumor tissue
Normal Brain Tissue
Cerebral Cortex
Glioma Patient 1 tumor tissue
Glioma Patient 2 tumor tissue
SUMO1
SUMO2

FIGURE S6

SUMO isoforms expression in pro-tumorigenic GBM immune cells

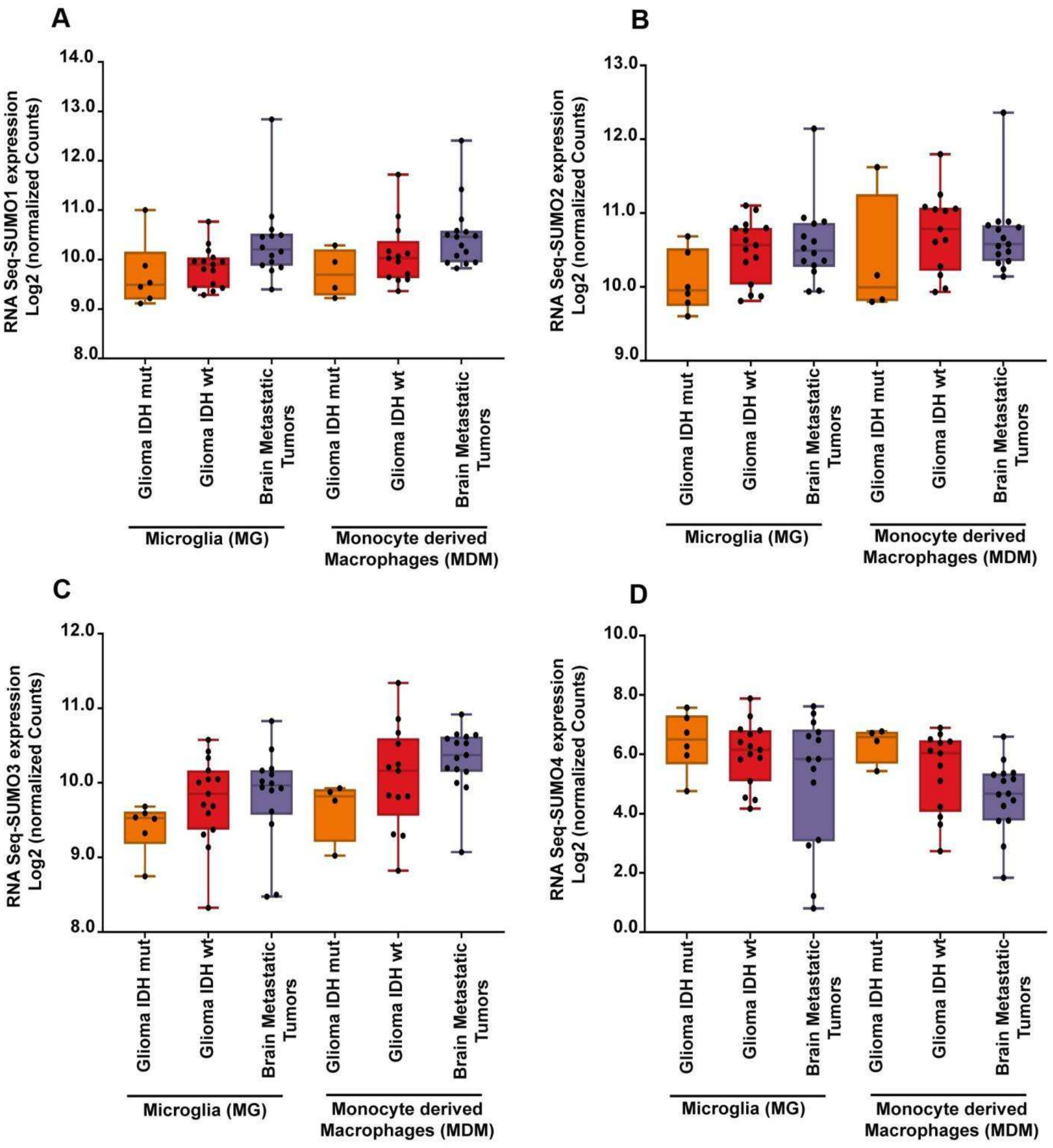

FIGURE S7

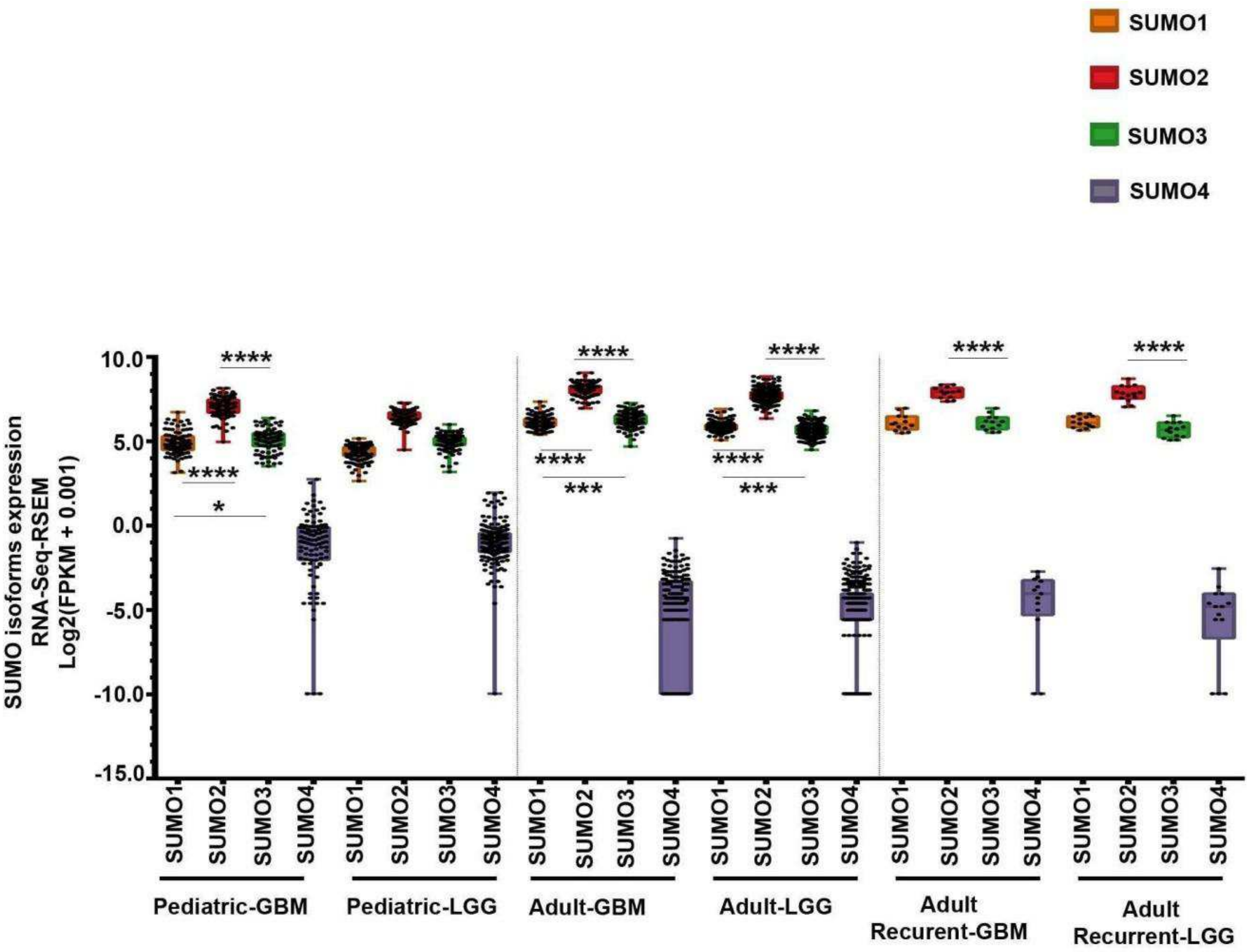
SUMO1
SUMO2
SUMO3
SUMO4
SUMO isoforms expression
RNA-Seq-RSEM
Log2(FPKM + 0.001)
10.0
5.0
0.0
-5.0
-10.0
-15.0
****
*
***
Pediatric-GBM
Pediatric-LGG
Adult-GBM
Adult-LGG
Adult
Recurent-GBM
Adult
Recurrent-LGG

FIGURE S8

Developing Human Brain (Pre-natal stages 8-37 pcw) [Allen Brain Atlas Data]

RNA-Seq

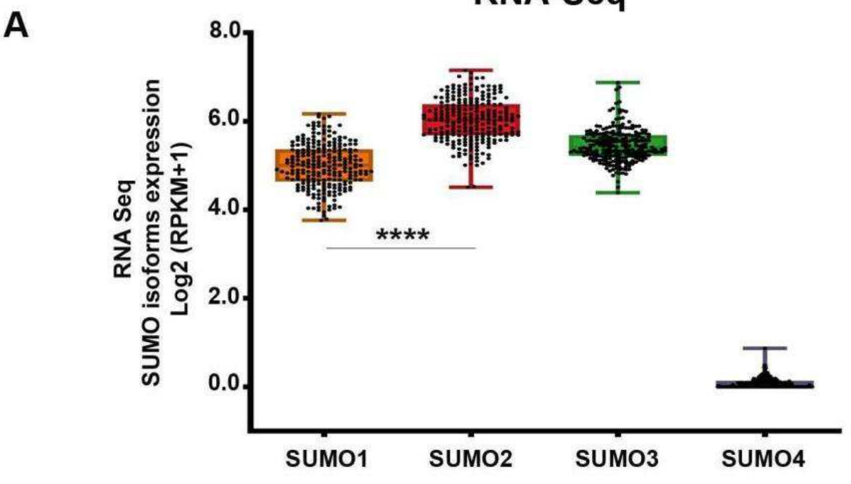

Developing Mouse Brain (Embryonic stage 14.5 dpc) [EMAGE DATA]

In situ hybridization

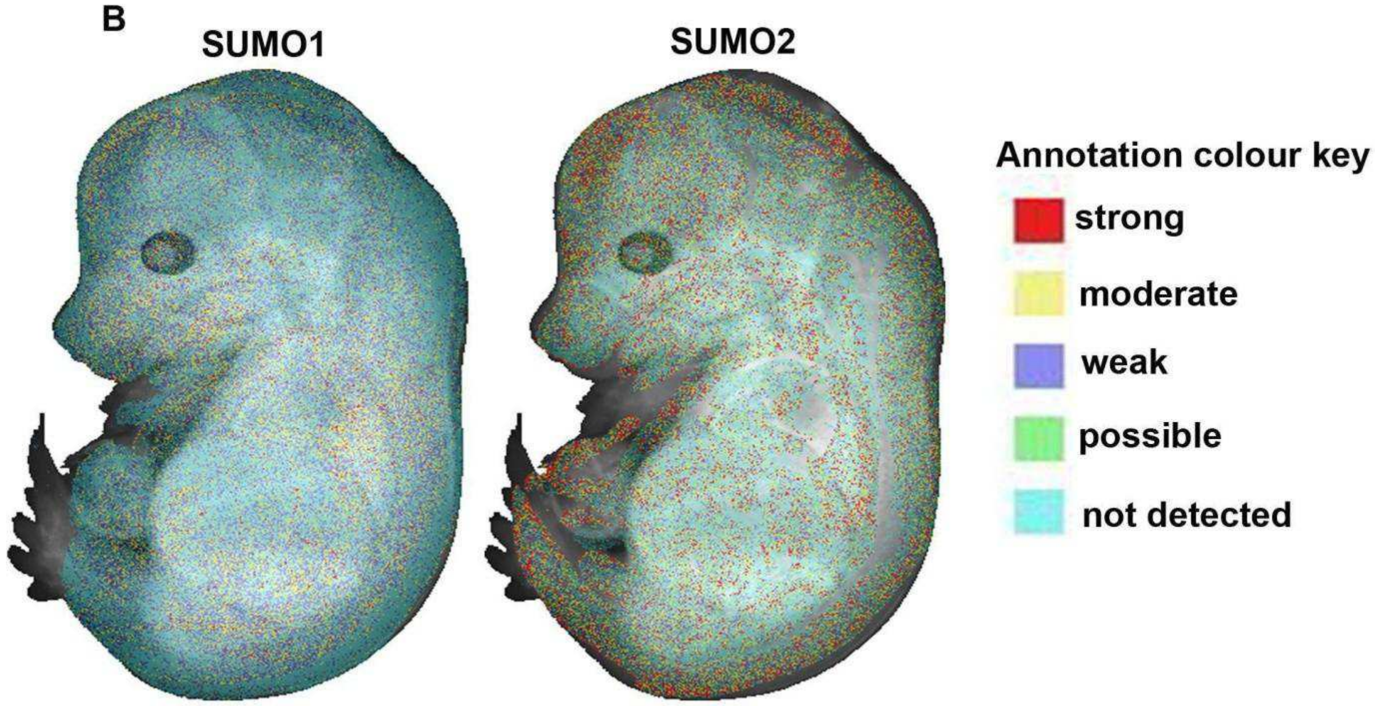

FIGURE S9

Cancer Cell Line Encyclopedia (CCLE)-SUMO isoform expression in various cancer cell lines

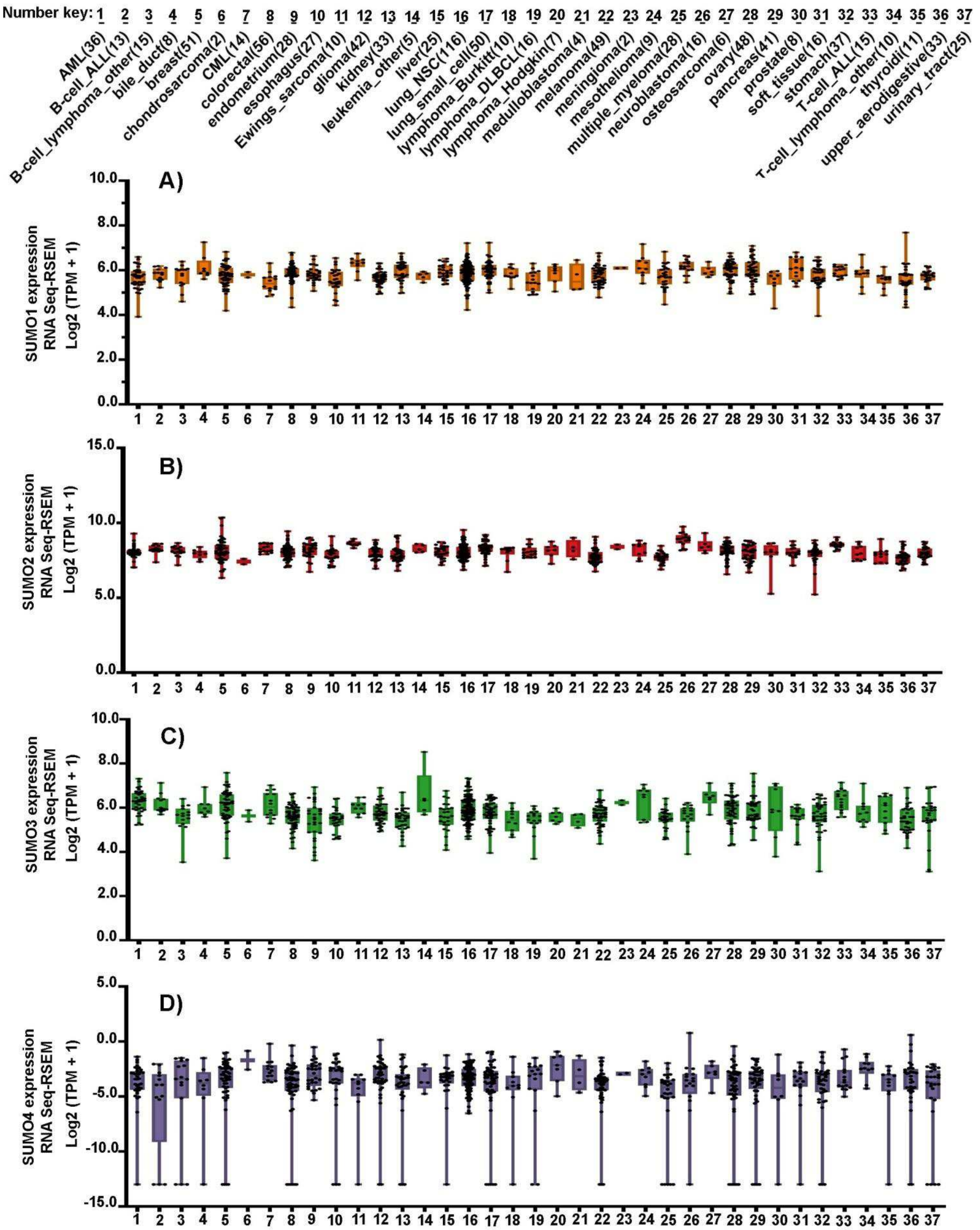

FIGURE S10

SUMO2 EXPRESSION ASSOCIATION WITH BREAST CANCER KAPLAN-MEIER SURVIVAL PLOT

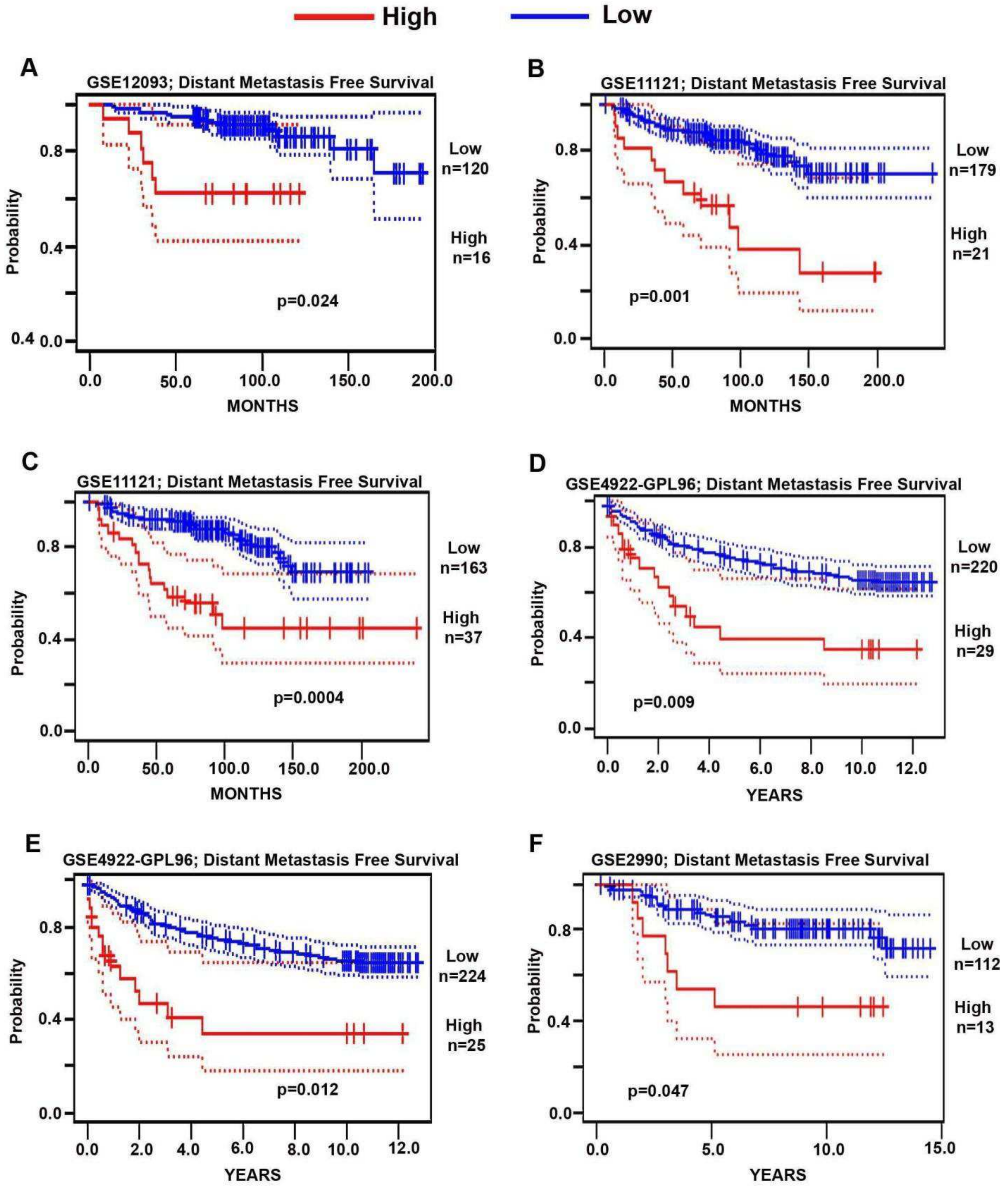

FIGURE S11

SUMO2 EXPRESSION ASSOCIATION WITH CANCER -KAPLAN-MEIER SURVIVAL PLOT

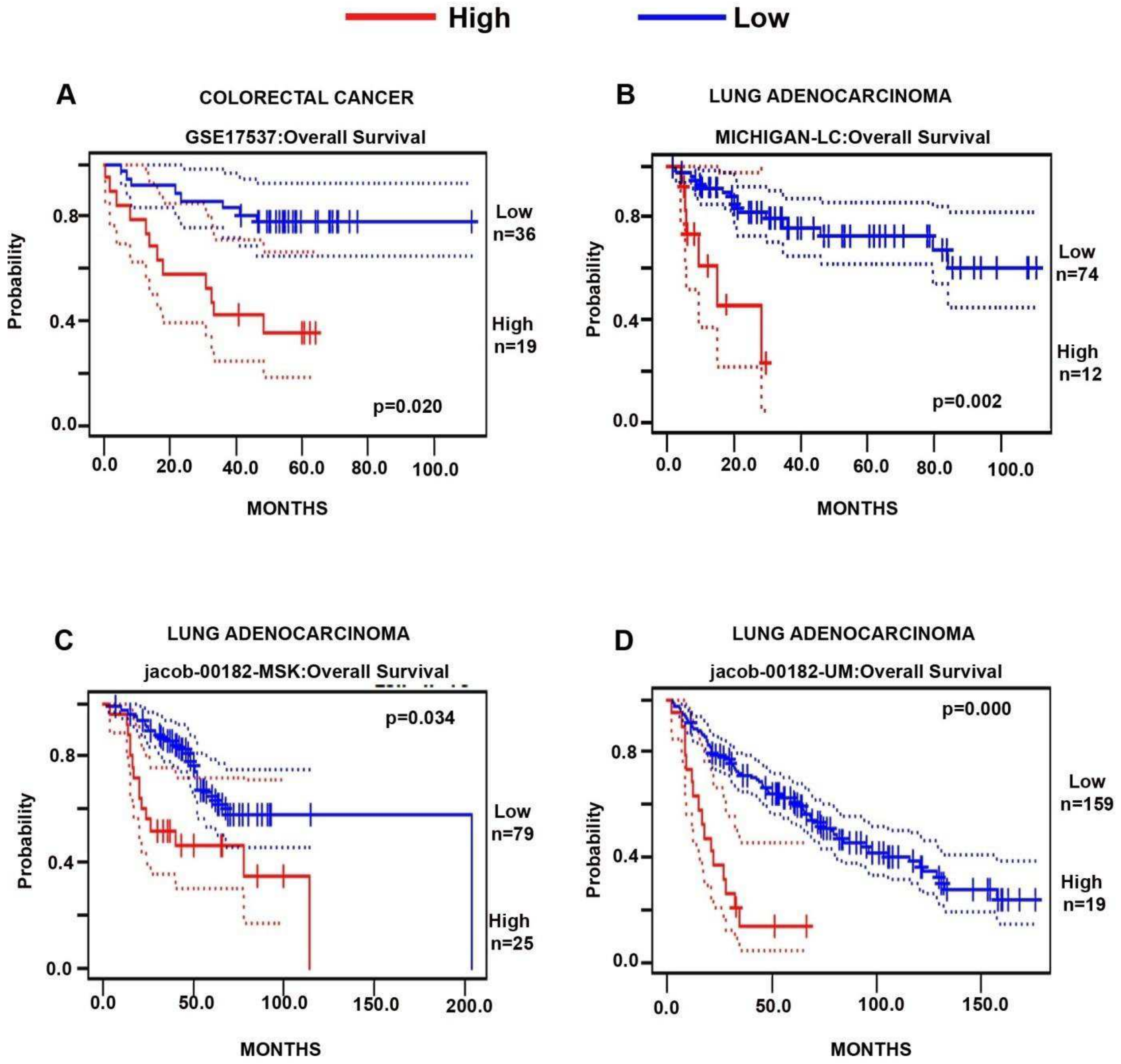

FIGURE S12
